# Active DHEA uptake in the prostate gland correlates with aggressive prostate cancer

**DOI:** 10.1172/JCI171199

**Published:** 2023-12-15

**Authors:** Xuebin Zhang, Zengming Wang, Shengsong Huang, Dongyin He, Weiwei Yan, Qian Zhuang, Zixian Wang, Chenyang Wang, Qilong Tan, Ziqun Liu, Tao Yang, Ying Liu, Ruobing Ren, Jing Li, William Butler, Huiru Tang, Gong-Hong Wei, Xin Li, Denglong Wu, Zhenfei Li

**Affiliations:** 1 State Key Laboratory of Cell Biology, CAS Center for Excellence in Molecular Cell Science, Shanghai Institute of Biochemistry and Cell Biology, and; 2 CAS Key Laboratory of Computational Biology, Shanghai Institute of Nutrition and Health, Chinese Academy of Sciences, University of Chinese Academy of Sciences, Shanghai, China.; 3 Department of Urology, Tongji Hospital, School of Medicine, Tongji University, Shanghai, China.; 4 Fudan University Shanghai Cancer Center and MOE Key Laboratory of Metabolism and Molecular Medicine, Department of Biochemistry and Molecular Biology, School of Basic Medical Sciences, Shanghai Medical College of Fudan University, Shanghai, China.; 5 Shanghai Key Laboratory of Metabolic Remodeling and Health, Institute of Metabolism and Integrative Biology, Fudan University, Shanghai, China.; 6 School of Medicine, The Chinese University of Hong Kong, Shenzhen, China.; 7 Department of Bioinformatics, Center for Translational Medicine, Second Military Medical University, Shanghai, China.; 8 Department of Pathology, Duke University School of Medicine, Durham, North Carolina, USA.; 9 State Key Laboratory of Genetic Engineering, School of Life Sciences, Human Phenome Institute, Metabonomics and Systems Biology Laboratory at Shanghai International Centre for Molecular Phenomics, Zhongshan Hospital, Fudan University, Shanghai, China.; 10 Key Laboratory of Systems Health Science of Zhejiang Province, School of Life Science, Hangzhou Institute for Advanced Study, University of Chinese Academy of Sciences, Hangzhou, China.

**Keywords:** Oncology, Drug screens, Prostate cancer, Ubiquitin-proteosome system

## Abstract

Strategies for patient stratification and early intervention are required to improve clinical benefits for patients with prostate cancer. Here, we found that active DHEA utilization in the prostate gland correlated with tumor aggressiveness at early disease stages, and 3βHSD1 inhibitors were promising for early intervention. [^3^H]-labeled DHEA consumption was traced in fresh prostatic biopsies ex vivo. Active DHEA utilization was more frequently found in patients with metastatic disease or therapy-resistant disease. Genetic and transcriptomic features associated with the potency of prostatic DHEA utilization were analyzed to generate clinically accessible approaches for patient stratification. UBE3D, by regulating 3βHSD1 homeostasis, was discovered to be a regulator of patient metabolic heterogeneity. Equilin suppressed DHEA utilization and inhibited tumor growth as a potent 3βHSD1 antagonist, providing a promising strategy for the early treatment of aggressive prostate cancer. Overall, our findings indicate that patients with active prostatic DHEA utilization might benefit from 3βHSD1 inhibitors as early intervention.

## Introduction

Prostate cancer is the most common cancer malignancy in men in Western countries, and its incidence is increasing rapidly in China (1, 2). Therapy resistance is inevitable due to accumulated mutations and the heterogeneous nature of advanced prostate cancer (3). Mortality from prostate cancer has declined only mildly, even after the approval of abiraterone and enzalutamide (4, 5). Considering the limited number of cancer cells and the infrequency of mutations at early disease stages, identification of patients potentially at risk for aggressive prostate cancer and development of related therapeutic or even preventive methods prior to androgen deprivation therapy (ADT) might benefit patients more substantially.

A deleterious androgenic environment accelerates the onset and early development of aggressive prostate cancer, which has been well substantiated by human genetics and ADT efficacy (6, 7). Identifying features of such a deleterious androgenic environment would facilitate patient stratification and drug development. Recently, we have traced steroidogenesis ex vivo in 524 fresh prostatic biopsy specimens collected from 241 patients and found that dehydroepiandrosterone (DHEA) is an important androgen precursor for the prostate gland physiologically (8). Enhanced DHEA metabolism has been reported to sustain the progression of castration-resistant prostate cancer (9). However, the clinical significance of prostatic DHEA metabolism at early disease stages has not been investigated.

The enzyme 3β-hydroxysteroid dehydrogenase type 1 (3βHSD1) catalyzes the rate-limiting step for the conversion of DHEA to dihydrotestosterone (DHT) (10, 11). Patients with homozygous *HSD3B1* (*1245C*) alleles, encoding 3βHSD1 (367T) isoform with persistent activity, had worse response to ADT, abiraterone, and enzalutamide treatment (12–15). Although the correlation of 3βHSD1 with tumor aggressiveness has not been investigated at early disease stages, these findings support a potential correlation of DHEA metabolism with the onset and development of aggressive prostate cancer. Recent advances regarding 3βHSD1 inhibitors support the promising potential of 3βHSD1 as a therapeutic target in the clinic (16, 17). DHEA metabolism–based patient stratification would benefit both diagnosis and disease therapy.

Unfortunately, the 3βHSD1 genotype fails to predict tumor aggressiveness in East Asian populations because of its low frequency, indicating racial heterogeneity of prostate cancer (17, 18). 3βHSD1 activity is not solely determined by its genotype (16, 19). Clinically accessible approaches, suitable for different genetic backgrounds, to comprehensively evaluate prostatic DHEA metabolism and 3βHSD1 activity could improve patient stratification and shed light on disease early intervention.

To find clues for early treatment of prostate cancer, we here retrospectively analyzed patient prostatic steroidogenesis in different clinical scenarios to discover potential metabolic features associated with aggressive prostate cancer. The genomic and transcriptional disparities associated with the metabolic features were further investigated to facilitate patient stratification across different genetic backgrounds. The mechanisms underlying patient metabolic heterogeneity were determined, and 3βHSD1 inhibitors were screened to treat patients with active prostatic DHEA utilization.

## Results

### Prostatic metabolic features associated with tumor aggressiveness.

**Figure 1:**
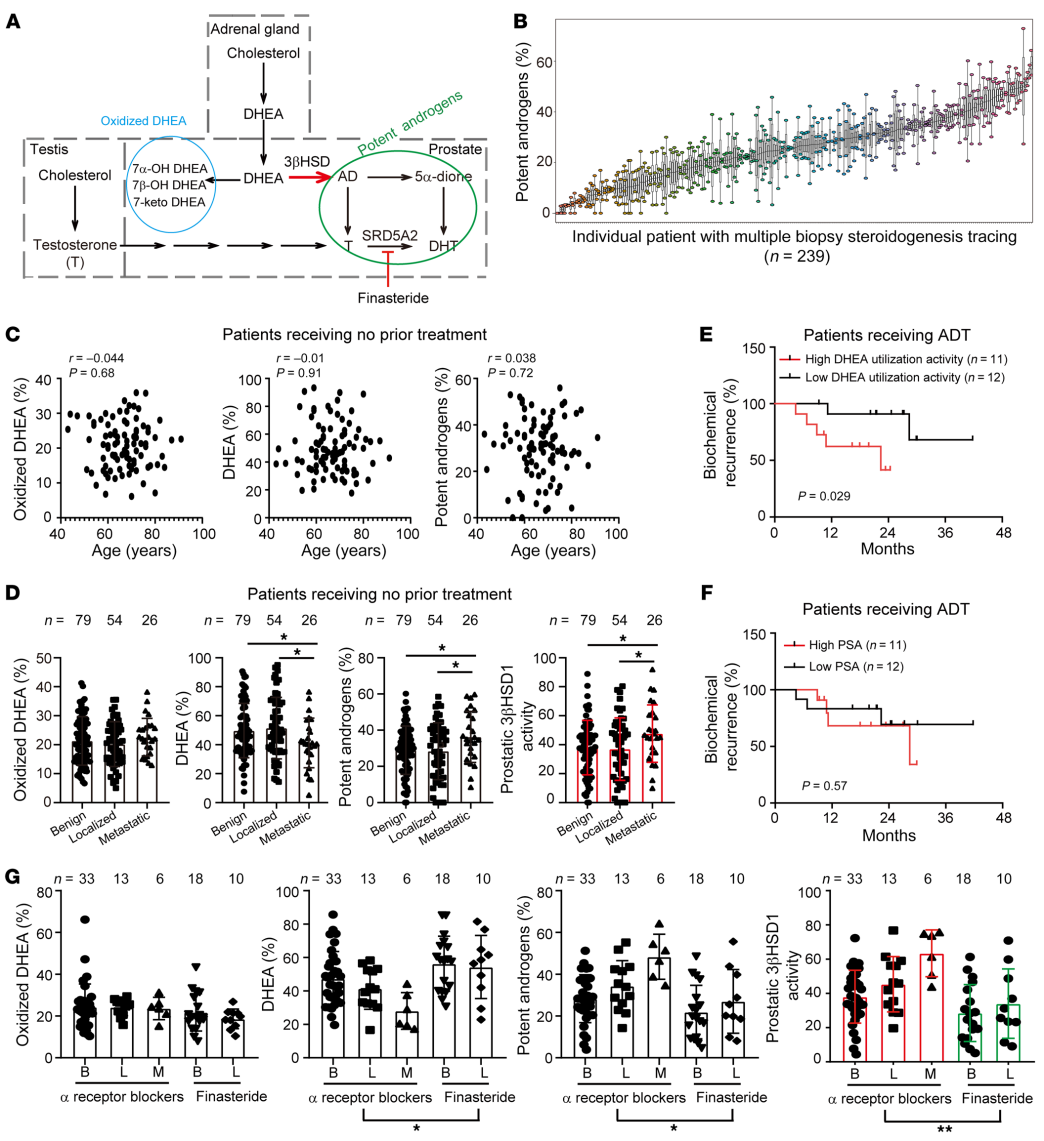
Correlations of prostatic DHEA utilization with tumor aggressiveness. (**A**) Schema of steroidogenesis in patient prostatic biopsies. (**B**) Intra- and interpatient heterogeneity in prostatic DHEA utilization. Multiple biopsies were collected from 239 patients for steroidogenesis tracing; *x* axis shows individual patients. Boxes show the interquartile range, and whiskers represent the minimum and maximum value. (**C**) Correlations of prostatic DHEA utilization activity with age. (**D**) Enhanced prostatic DHEA utilization in metastatic patients. All patients were naive to treatment. Prostatic 3βHSD1 activity is calculated as potent androgens/(DHEA + potent androgens) × 100%. Bars, median; lines, interquartile range. Two-tailed Student’s *t* test. (**E**) Association of prostatic DHEA utilization with duration of response to ADT. Biopsies were collected prior to ADT. Log-rank test. (**F**) Baseline PSA could not predict ADT response. Log-rank test. (**G**) Suppressed prostatic DHEA utilization in finasteride-treated patients. Two-way ANOVA. *P < 0.05; **P < 0.01.

To determine the prostatic metabolic features associated with tumor aggressiveness, fresh prostatic biopsy samples were transiently cultured ex vivo and treated with [^3^H]-DHEA (8). Androgen metabolites were separated and analyzed by HPLC–β-RAM system at different time points (20). DHEA was observed to be actively converted to potent androgens (including androstenedione, testosterone, DHT, and 5α-androstanedione) as well as oxidized DHEA (including 7α-OH DHEA, 7β-OH DHEA, and 7-keto DHEA) in biopsy specimens (Figure 1A) (8, 10). Because oxidized DHEA could not activate androgen receptor (AR) signaling, the potency of DHEA utilization was evaluated based on the generation of potent androgens. Enzyme activity in biopsies was evaluated by calculation of the ratio between different metabolites. For example, prostatic 3βHSD1 activity was assessed as (concentration of potent androgens)/(concentration of DHEA + potent androgens). Although the potency of DHEA utilization diverged across individuals, it was highly concordant within individuals and did not decline by age (Figure 1, B and C) (8). Previously, we showed that steroidogenesis in different biopsies from the same patient is not markedly affected by factors such as cancer cell content, cell types, Gleason score, and anatomic structures (8). Thus, it is feasible to evaluate patient prostatic DHEA utilization with 1 or 2 prostatic biopsies from the same individual.

The clinical significance of prostatic DHEA utilization was then retrospectively analyzed in different clinical scenarios. Results of 159 prostatic biopsy samples from 85 patients who had elevated prostate-specific antigen (PSA) and were naive to treatment demonstrated that enhanced DHEA utilization was more frequently found in biopsies from patients with metastatic prostate cancer, as indicated by the generation of potent androgens and prostatic 3βHSD1 activities (Figure 1D and Supplemental Table 1; supplemental material available online with this article; https://doi.org/10.1172/JCI171199DS1). Biopsies were collected from 23 patients at the baseline of ADT, and the metabolic results showed that patients with enhanced prostatic DHEA utilization exhibited a shorter treatment duration (Figure 1E and Supplemental Table 2). In contrast, baseline PSA failed to predict the response to ADT in these 23 patients (Figure 1F). Together, these data demonstrate the association of prostatic DHEA utilization with tumor aggressiveness.

Finasteride and dutasteride, inhibitors of steroid 5α-reductase (SRD5A), have been reported to reduce the incidence of prostate cancer (21, 22). Twenty-eight biopsies were collected from 14 finasteride-treated patients and 52 biopsies from 27 patients treated with α-receptor blockers for benign prostatic hyperplasia. Among α-receptor blocker–treated patients, enhanced DHEA utilization was also observed in biopsies from metastatic patients (Figure 1G and Supplemental Table 3). Biopsies from finasteride-treated patients obtained suppressed DHEA utilization compared with biopsies from α-receptor blocker–treated patients (Figure 1G). Finasteride had a long half-life in patients (23). To exclude the existence of residual finasteride in biopsies, the percentage of androstenedione (AD), which should be accumulated with the existence of residual finasteride, was analyzed. Biopsies from finasteride-treated patients generated a similar amount of AD compared with the other 2 groups, indicating no residual finasteride in biopsies (Supplemental Figure 1A). Finasteride and dutasteride also failed to directly regulate DHEA utilization in LNCaP cells and biopsy samples (Supplemental Figure 1, B and C). The suppressed prostatic DHEA utilization and 3βHSD1 activity might explain the low incidence of prostate cancer in finasteride-treated patients. Together, these data indicate the association of active prostatic DHEA utilization with tumor occurrence.

### Genomic signature reflecting prostatic DHEA utilization.

**Figure 2:**
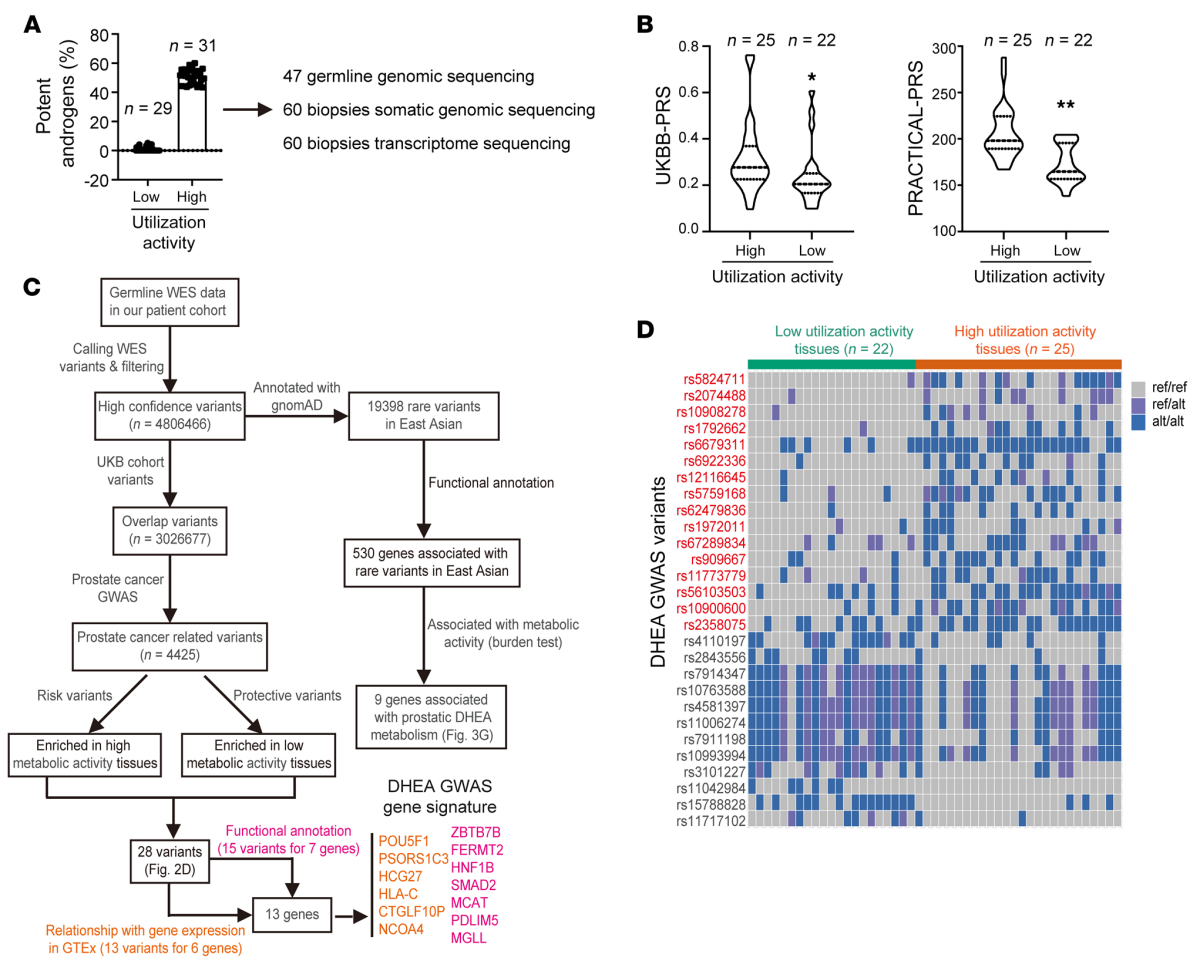
Generation of GWAS signature associated with prostatic DHEA utilization. (**A**) Sixty biopsies from 47 patients with distinct DHEA metabolic features were selected for sequencing. Somatic genomic DNA and RNA were extracted from 60 biopsies. Germline DNA was extracted from 47 patients with blood samples. (**B**) UKBB-PRS and PRACTICAL-PRS in patients with enhanced and suppressed prostatic DHEA utilization, respectively. Two-tailed Student’s *t* test. (**C**) Flowchart for screening prostatic DHEA utilization–associated variants. WES, whole-exome sequencing. (**D**) List of variants associated with prostatic DHEA utilization features. Risk variants are shown in red and protective variants in black. Ref, reference allele; alt, alternative allele. *P < 0.05; **P < 0.01.

Tracing prostatic steroidogenesis ex vivo to evaluate tumor aggressiveness is labor-intensive and time-consuming. To develop clinically feasible approaches, whole-exome sequencing and transcriptome sequencing were performed with blood and biopsy samples from patients with distinct DHEA metabolism features. Considering that about 10% of prostate cancer will develop into aggressive prostate cancer, 31 biopsies from 25 patients (prostatic 3βHSD1 activity showing the top 10% in our cohort) showing enhanced prostatic DHEA utilization and 29 biopsies from 22 patients (prostatic 3βHSD1 activity showing the bottom 10% in our cohort) showing limited DHEA utilization were selected for further analysis (Figure 2A).

**Figure 3:**
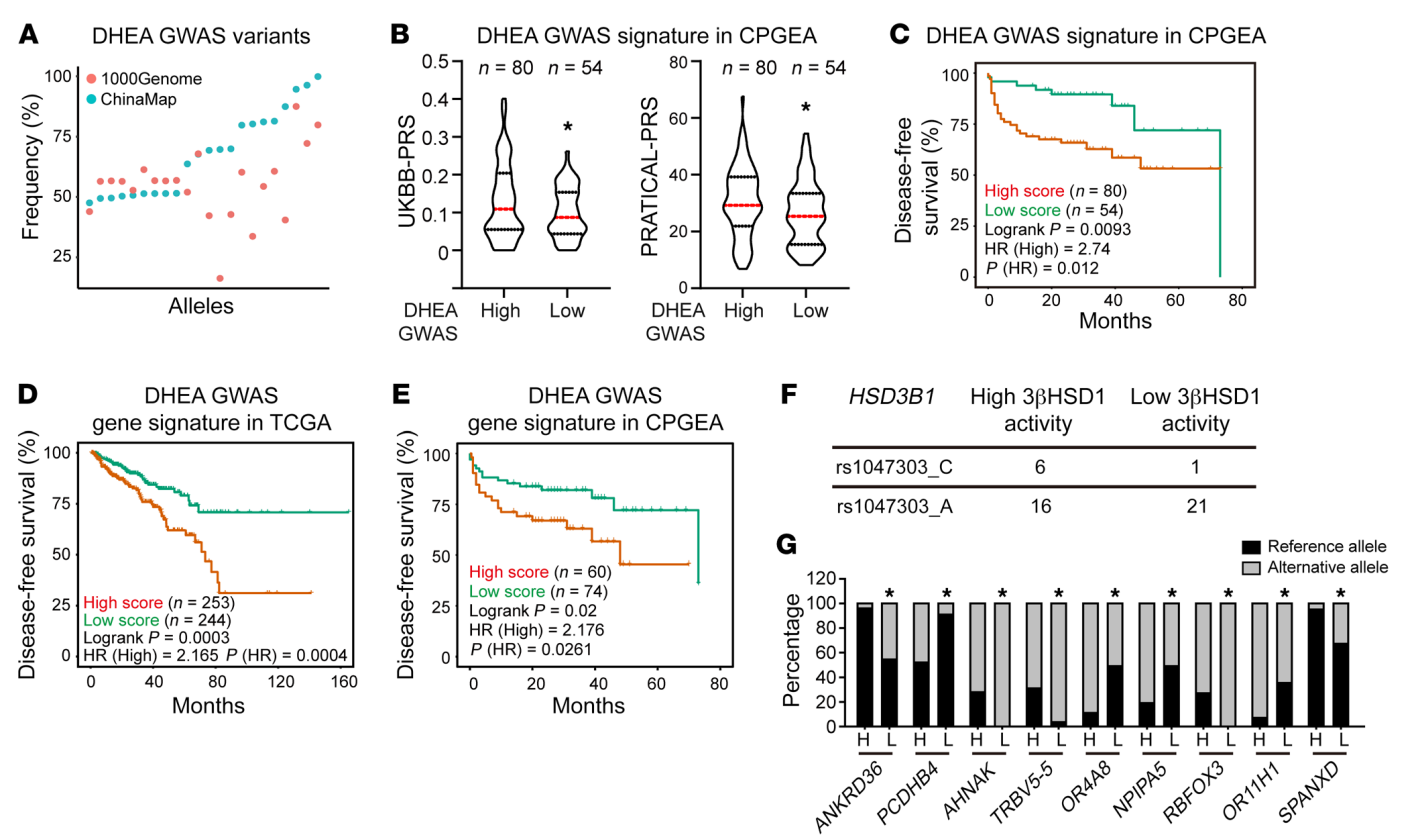
Characterization of GWAS signature associated with prostatic DHEA utilization. (**A**) Frequency of DHEA GWAS variants in Chinese and European populations. Minor allele frequency of variants was determined with 1000 Genomes and ChinaMAP databases. (**B** and **C**) Validation of DHEA GWAS signature in CPGEA. Prostate cancer risk and treatment duration were different in patients with distinct scores of DHEA GWAS signature. One-tailed Student’s *t* test for **B**; log-rank test for **C**. (**D** and **E**) Genes associated with DHEA GWAS signature for patient stratification in TCGA and CPGEA. Genes associated with variants were determined using GTEx and functional annotation. (**F**) Frequency of *HSD3B1* variants in our cohort. (**G**) Uncommon variants and related genes showing different distributions in patients with distinct prostatic DHEA utilization features. H, high–metabolic activity tissues; L, low–metabolic activity tissues. Burden test was applied for significance calculation. *P < 0.05.

Germline genetic disparity associated with prostatic DHEA utilization was first assessed. Different polygenic risk scores (PRSs) have been developed to describe genetic predisposition of prostate cancer risk, including UK Biobank (UKBB)–PRS and PRACTICAL-PRS (24). In our cohorts, higher UKBB-PRS and PRACTICAL-PRS were observed in patients with enhanced prostatic DHEA utilization, indicating a higher risk of prostate cancer incidence in these patients (Figure 2B). To generate a genome-wide association (GWAS) signature reflecting prostatic DHEA utilization features across different populations, single-nucleotide polymorphisms (SNPs) identified in our cohort were crossed with prostate cancer–associated sites from UKBB and GWAS catalogs (Figure 2C) (25). Variants were further tested based on their relationship with prostatic metabolic features. A total of 28 SNPs associated with prostate cancer incidence and enhanced prostatic DHEA utilization were selected to generate a DHEA GWAS signature (Figure 2, C and D). The frequencies of these SNPs were comparable in different populations, making it a candidate set for assessing genetic risk of tumor aggressiveness in different ethnic groups (Figure 3A). The predictive capability of the DHEA GWAS signature, composed of 28 SNPs, was further validated in a cohort of the Chinese Prostate Cancer Genome and Epigenome Atlas (CPGEA), a comprehensive Chinese prostate cancer genomic database with over 1,200 genomic data sets from more than 200 pairs of primary prostate cancers and matched normal tissues (26). Twenty-seven DHEA GWAS variants were confirmed in CPGEA and then used for patient stratification. Individuals with high scores of the DHEA GWAS signature also showed higher UKBB-PRS and PRACTICAL-PRS, suggesting a shared genetic component of prostatic DHEA utilization and prostate cancer risk (Figure 3B). Furthermore, patients with higher DHEA GWAS signature scores were more resistant to ADT (Figure 3C). However, only 4 variants could be found in The Cancer Genome Atlas (TCGA) database. Thus, we investigated potential genes regulated by DHEA GWAS variants through linking with expression quantitative trait loci (eQTLs) identified in Genotype-Tissue Expression (GTEx) prostate tissues and variant effect predictor (VEP) functional annotation (Supplemental Figure 2A) (27, 28). A gene expression signature with a combination of these 13 genes successfully identified patients at risk after prostatectomy in a TCGA cohort of prostate cancer (Figure 3D). Consistently, this gene signature could also be used for patient stratification in CPGEA, supporting the clinical relevance of the DHEA GWAS signature (Figure 3E). *HSD3B1 (1245C)* allele, with a low frequency in East Asian populations, was also checked in our cohort. Seven patients with heterozygous *HSD3B1 (1245C)* were found in our cohort, but none were homozygous *HSD3B1 (1245C)*. Of these 7 patients, 6 showed enhanced prostate DHEA utilization, and 1 showed suppressed prostate DHEA utilization (Figure 3F). Furthermore, correlations of GWAS signature variants with *HSD3B1* genotype were found in the UKBB database. Patients with heterozygous and homozygous *HSD3B1 (1245C)* had enriched GWAS risk variants but less protective variants than patients with homozygous *HSD3B1 (1245A)* (Supplemental Figure 2B). To find uncommon variants like *HSD3B1 (1245C)* in our cohort, variants found in our cohort that are rare in the general population according to gnomAD allele frequency were tested for their correlation with gene expression and prostatic DHEA utilization (Figure 2B) (29). Nine genes enriched with uncommon variants were identified, which showed markedly different distributions in biopsies with distinct metabolic features (Figure 3G and Supplemental Table 4). Together, these data reveal germline alleles associated with prostatic DHEA utilization to indicate tumor onset and aggressiveness.

**Figure 4:**
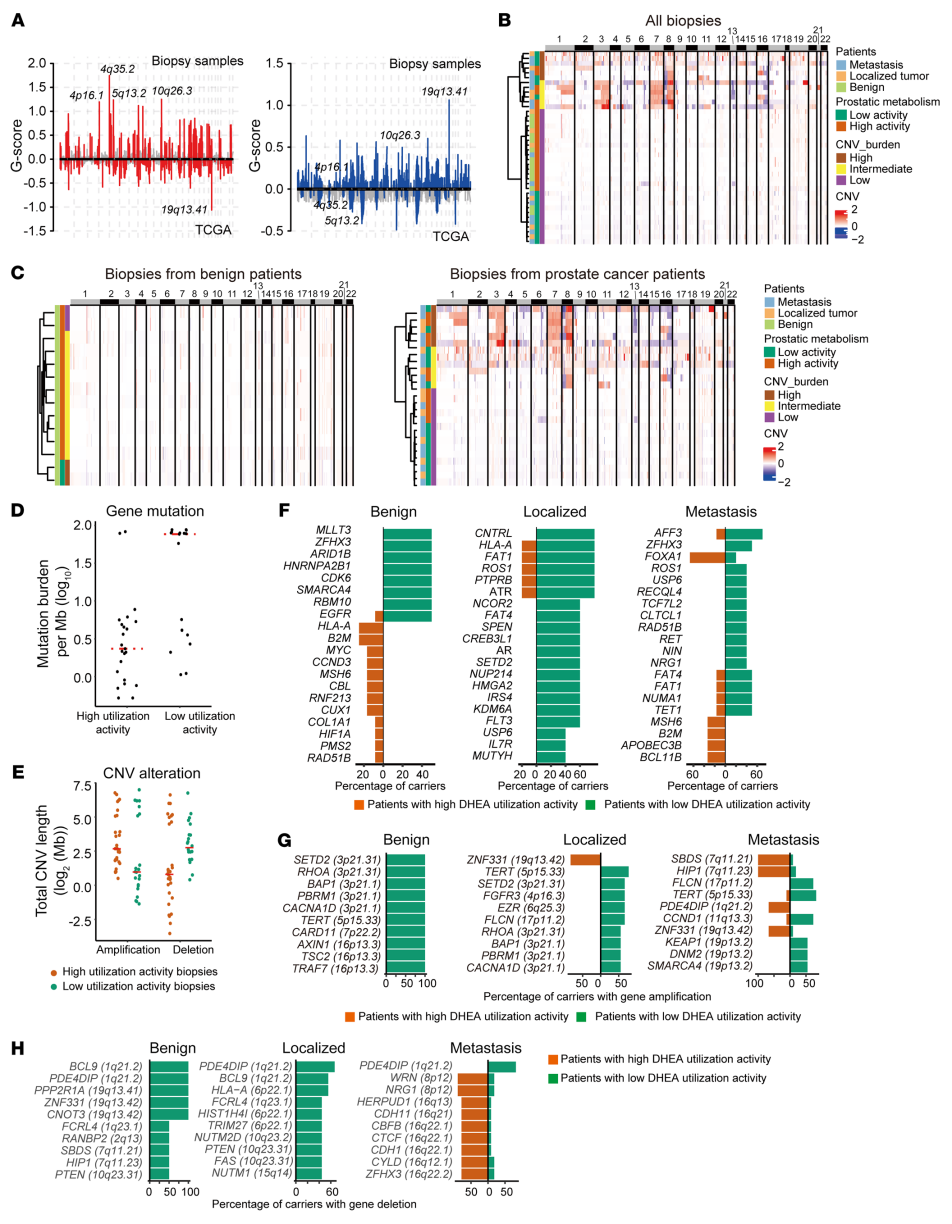
Somatic mutations associated with prostatic 3βHSD1 activity. (**A**) Genomic regions with marked recurrent somatic copy number variations (CNVs). Genomic DNA from biopsies was used for somatic mutation detection. (**B** and **C**) Heatmaps of somatic CNV burdens in biopsies. Results of all biopsies are shown in **B**, and separately displayed as benign patients and cancer patients in **C**. (**D**) Mutation burdens in biopsies with different metabolic features. (**E**) CNV in biopsies with different metabolic features. (**F**) Most frequent mutated genes in patients with different metabolic features at different disease stages. A patient would be identified as a carrier when a mutation was detected (regardless of its alternative allele fraction) in any of his biopsy samples, and the percentage of carriers was calculated in patients at different disease stages. (**G**) Most amplified genes in patients with different metabolic features at different disease stages. (**H**) Most deleted genes in patients with different metabolic features at different disease stages.

We next analyzed somatic mutations associated with prostatic DHEA utilization. Substantial genomic alterations were observed in the genome of biopsy samples (Figure 4A). However, the copy number variation burdens were mainly associated with disease stages but not with prostatic DHEA metabolic features (Figure 4B). Biopsies from benign patients showed limited genomic alterations, while biopsies from patients with prostate cancer exhibited more alterations (Figure 4C and Supplemental Figure 3A). However, we still found that biopsies with suppressed DHEA utilization obtained higher mutation load and gene deletion frequency, but lower gene amplification frequency, indicating a potential correlation between genomic alterations and androgenic environment (Figure 4, D and E).

To find DHEA utilization–related pretumorous somatic mutations that might give rise to malignant clones in later stages, a patient would be identified as a mutation carrier when a mutation was detected (regardless of its alternative allele fraction) in any of his biopsy samples, and the percentage of carriers was calculated in patients at different disease stages. Notably, somatic mutations in benign samples are of low alternative allele fraction (typically <0.05), representing sporadic clonal expansions in prostate glands (Supplemental Figure 3B). However, patients with different metabolic features might have different gene mutations enriched. For example, *MSH6* and *B2M* were more frequently mutated in patients with enhanced DHEA utilization, while *USP6* and *ZFHX3* were more frequently mutated in patients with suppressed DHEA utilization (Figure 4F). Similar strategies were applied to analyze gene amplification and deletion. Still, the genomic alteration was dramatically lower in benign patients than in patients with prostate cancer (Supplemental Figure 3, C and D). The region around 3p21 was more frequently amplified and the region around 1q21 was more frequently deleted in patients with low metabolic activity (Figure 4, G and H). *PTEN* deletion was more frequently found in patients with low metabolic activity (Figure 4H). Interestingly, *PDE4DIP* was exclusively amplified in patients with high metabolic activity but deleted in patients with low metabolic activity; *ZNF331* showed an almost contrary distribution, with detailed mechanisms to be further investigated (Figure 4, G and H). These results indicate that patients with low metabolic activity have distinct genomic alterations, presumably to increase tumor cell survival independent of androgens.

### Transcriptomic signature reflecting prostatic DHEA utilization.

**Figure 5:**
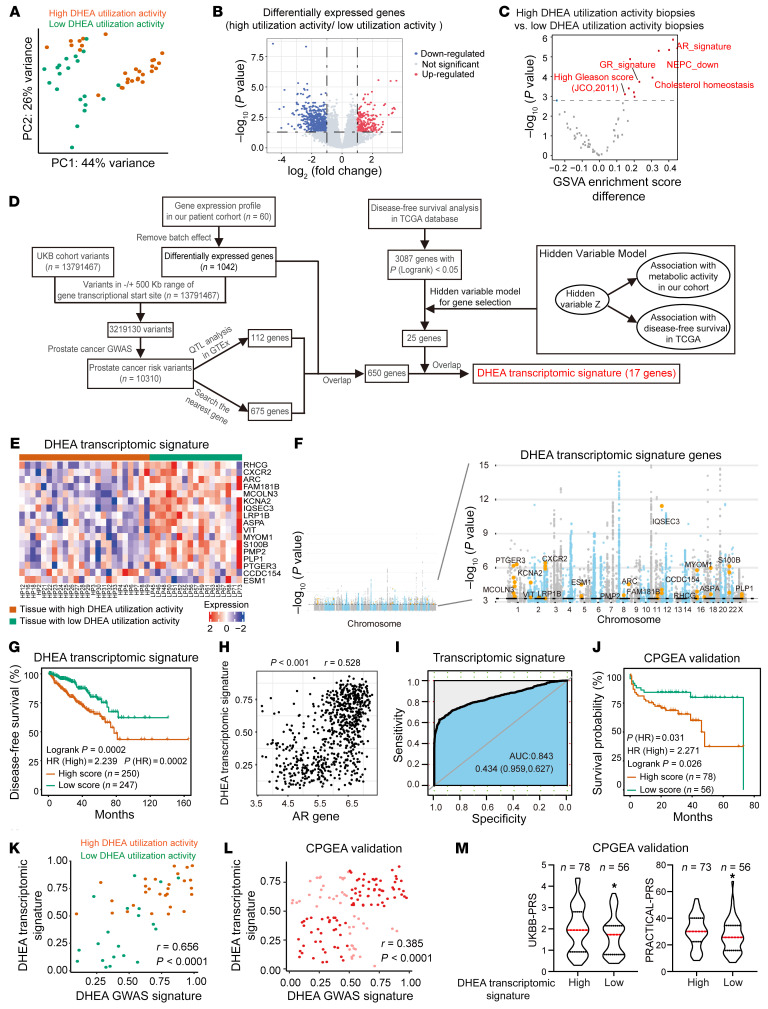
Transcriptomic signature reflecting prostatic DHEA utilization. (**A**) Principal component analysis on biopsy transcriptome. Red, biopsies with high metabolic activity; blue, biopsies with low metabolic activity. (**B**) Differentially expressed genes in biopsies with high or low metabolic activity. (**C**) Volcano plot for the differential expression analysis at pathway level between biopsies with different metabolic activity. Gene set variation analysis (GSVA) was performed according to hallmark gene sets. (**D**) Flowchart for the generation of transcriptomic signature reflecting prostatic DHEA utilization. (**E**) Heatmap of DHEA transcriptomic signature genes in biopsies. (**F**) Prostate cancer GWAS hits on DHEA transcriptomic signature genes. Prostate cancer GWAS summary statistics were determined according to UK Biobank and are shown in gray and blue. Hits on signature genes are shown in yellow. (**G**) DHEA transcriptomic signature for patient stratification in TCGA. Log-rank test. (**H**) Pearson’s correlations of DHEA transcriptomic signature with AR-responsive hallmark in TCGA and GTEx databases. Pearson’s correlation test. (**I**) Validation of DHEA transcriptomic signature in prediction of biopsy metabolic activities. Random grouping has been performed over 200 times by selection of 14 biopsies with high metabolic activity and 10 biopsies with low metabolic activity from the 60 sequenced biopsies. (**J**) DHEA transcriptomic signature for patient stratification in CPGEA. Log-rank test. (**K** and **L**) Correlations of DHEA transcriptomic signature genes with DHEA GWAS signature in our cohort (**K**) and CPGEA (**L**). Pearson’s correlation coefficient for the correlation analysis. (**M**) Genetic features associated with DHEA transcriptomic signature genes in CPGEA. One-tailed Student’s *t* test. *P < 0.05.

Transcriptome sequencing was also performed with these biopsies. Biopsies with higher metabolic activity showed distinct transcriptomic features compared with those with lower metabolic activity (Figure 5A). Differentially expressed genes associated with prostatic DHEA utilization were identified (Figure 5B and Supplemental Figure 4A). Consistent with previous reports, biopsies with high metabolic activity showed activated AR and glucocorticoid receptor signaling but suppressed neuroendocrine prostate cancer signature (Figure 5C and Supplemental Figure 4B) (30, 31). Tumor aggressiveness associated with Gleason score grade signature was also enriched in biopsies with high metabolic activity (Figure 5C and Supplemental Figure 4B) (32). To generate a gene expression signature reflecting the association between prostatic DHEA utilization and tumor aggressiveness in different ethnicities, differentially expressed genes in our cohort were compared with prostate cancer risk genes identified in the UKBB cohort and GWAS catalogs. A latent variable hierarchical model was used to train a transcriptomic signature using the TCGA database, with a hidden variable layer representing prostatic metabolic status (Figure 5D). A total of 17 genes were selected as DHEA transcriptomic signature. These genes showed different expression in biopsies of different metabolic features and correlated with prostate cancer risk (Figure 5, E and F). Moreover, the DHEA transcriptomic signature successfully stratified patients with different treatment durations or diseases stages and correlated with tumor stage, Gleason score, and lymph node metastasis in TCGA (Figure 5G and Supplemental Figure 5). A positive correlation of DHEA transcriptomic signature and AR pathway was observed in the UKBB and GTEx databases (Figure 5H). The predictive capability of the DHEA transcriptomic signature was further validated. The robustness of the correlations of DHEA transcriptomic signature with prostatic DHEA utilization was validated by random grouping. Fourteen biopsies with high metabolic activity and 10 biopsies with low metabolic activity were randomly selected over 200 times from the 60 sequenced biopsies. DHEA transcriptomic signature and individual genes clearly distinguished the high-activity biopsies from those with low activity (Figure 5I and Supplemental Figure 6). Consistently, we observed that patients with high DHEA transcriptomic signature scores had shorter treatment durations in the validation cohort of CPGEA (Figure 5J). The correlation between DHEA transcriptomic signature and DHEA GWAS signature was also analyzed. Patients with higher DHEA transcriptomic signature scores also displayed a high DHEA GWAS signature score in both our cohort and CPGEA (Figure 5, K and L). Consistent patterns were also observed between inferred DHEA status and genetic predisposition of prostate cancer, and patients with higher DHEA transcriptomic signature score in CPGEA also had higher PRACTICAL-PRS and UKBB-PRS (Figure 5M). Together, these data indicate that DHEA transcriptomic signature reflects prostatic metabolic features and predicts tumor aggressiveness.

### UBE3D-mediated 3βHSD1 ubiquitylation and metabolic heterogeneity.

**Figure 6:**
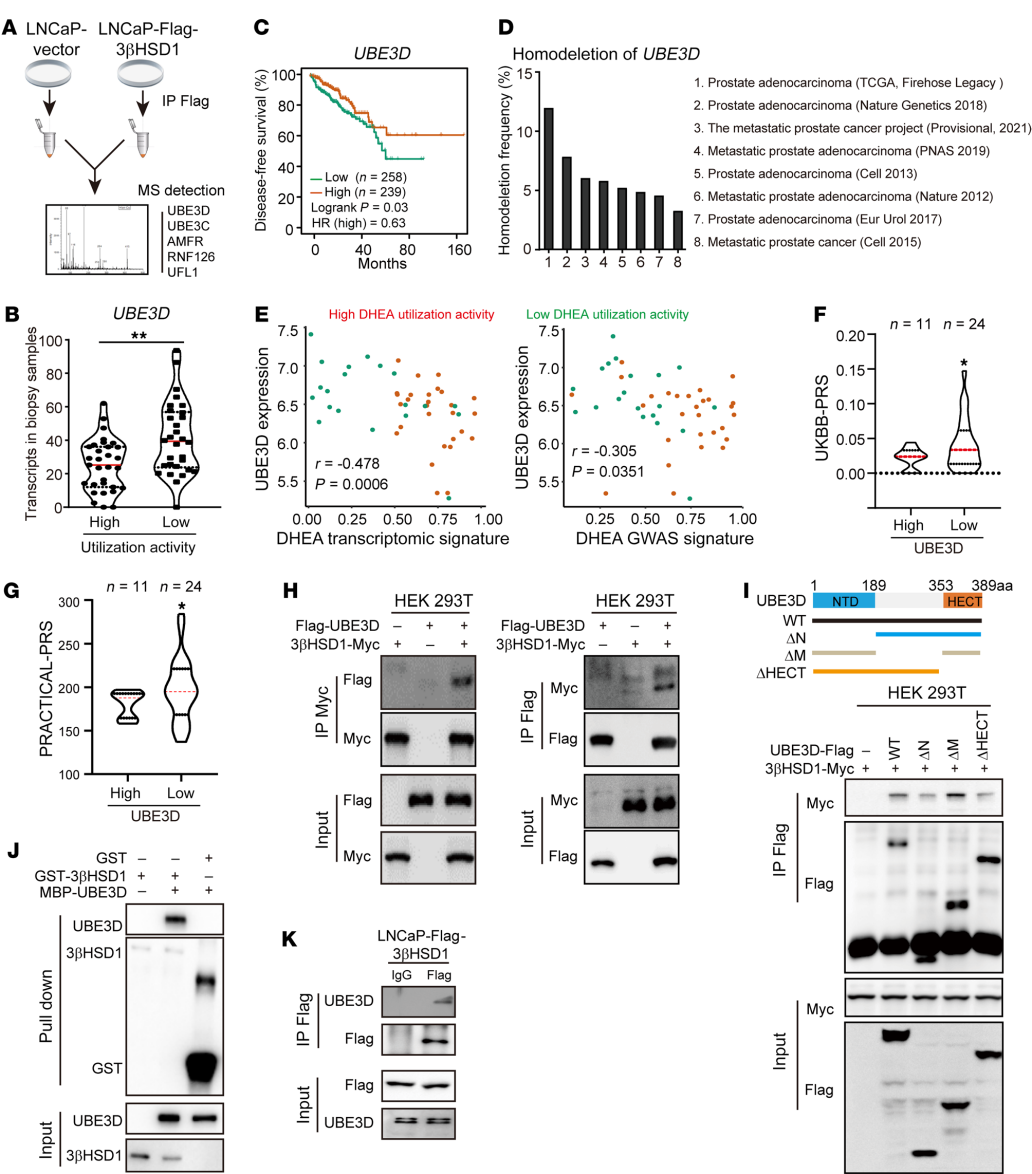
UBE3D binds to 3βHSD1. (**A**) Schema of potential ubiquitin ligase screening. Immunoprecipitation–mass spectrometry (IP-MS) was performed in LNCaP cells expressing FLAG-tagged 3βHSD1 (LNCaP-FLAG-3βHSD1). (**B**) UBE3D mRNA abundance in biopsies with high or low metabolic activities. (**C**) Correlation of UBE3D levels with treatment duration in TCGA. Log-rank test. (**D**) Deletion frequency of UBE3D in prostate cancer. (**E**) Correlation of UBE3D levels with DHEA transcriptomic signature and DHEA signature in our cohort. Pearson’s correlation. (**F** and **G**) UKBB-PRS and PRACTICAL-PRS in patients with different UBE3D levels. One-tailed Student’s *t* test. (**H**) Interaction between UBE3D and 3βHSD1. UBE3D and 3βHSD1 were overexpressed in HEK293T cells. (**I**) Interaction of 3βHSD1 with different UBE3D truncations. (**J**) Direct binding of UBE3D to 3βHSD1 in vitro. UBE3D was purified in *E*. *coli* system, and 3βHSD1 was purified in Sf9 cells. (**K**) Endogenous UBE3D binds to 3βHSD1. Stable cell line with FLAG-3βHSD1 expressed at comparable levels of endogenous 3βHSD1 was used. Two-tailed Student’s *t* test. *P < 0.05; **P < 0.01.

To further reveal the mechanisms underlying patient heterogeneity with respect to prostatic metabolic features, differentially expressed genes in biopsies with different metabolic features were cross-analyzed with 3βHSD1 interactome. LNCaP cells stably expressing FLAG-3βHSD1 (LNCaP-FLAG-3βHSD1) were used for immunoprecipitation–mass spectrometry (IP-MS) to find proteins interacting with 3βHSD1 (Figure 6A). Five ubiquitin ligases were identified to potentially bind to 3βHSD1. *UBE3D* was the only gene showing lower expression in biopsies with enhanced DHEA utilization (Figure 6B and Supplemental Figure 7A). Consistently, patients with lower UBE3D expression showed shorter treatment duration in the TCGA database, while the other 4 ligases exhibited no significant correlation with disease progression (Figure 6C and Supplemental Figure 7B). Notably, UBE3D deletion was frequently present in different cohorts, indicating that the correlation of UBE3D with prostate cancer is not limited to East Asians but involves different ethnicities (Figure 6D) (33). In our patient cohort, UBE3D expression negatively correlated with DHEA transcriptomic signature and DHEA GWAS signature (Figure 6E). Patients with low UBE3D expression had higher UKBB-PRS and PRACTICAL-PRS (Figure 6, F and G). Furthermore, 3βHSD1 abundance increased only when UBE3D or AMFR was knocked down in LNCaP cells (Supplemental Figure 7C). These results together indicate the negative correlation of UBE3D with prostatic 3βHSD1 activity.

The interaction between UBE3D and 3βHSD1 was further confirmed by co-IP assays (Figure 6H). Multiple domains of UBE3D, including HECT domain, interacted with 3βHSD1 (Figure 6I). UBE3D directly interacted with 3βHSD1 in vitro (Figure 6J). Because of poor antibody quality, the endogenous IP was performed in LNCaP-FLAG-3βHSD1 cells, and FLAG-3βHSD1 was observed to be bound to endogenous UBE3D (Figure 6K). These results support the interaction of UBE3D with 3βHSD1.

**Figure 7:**
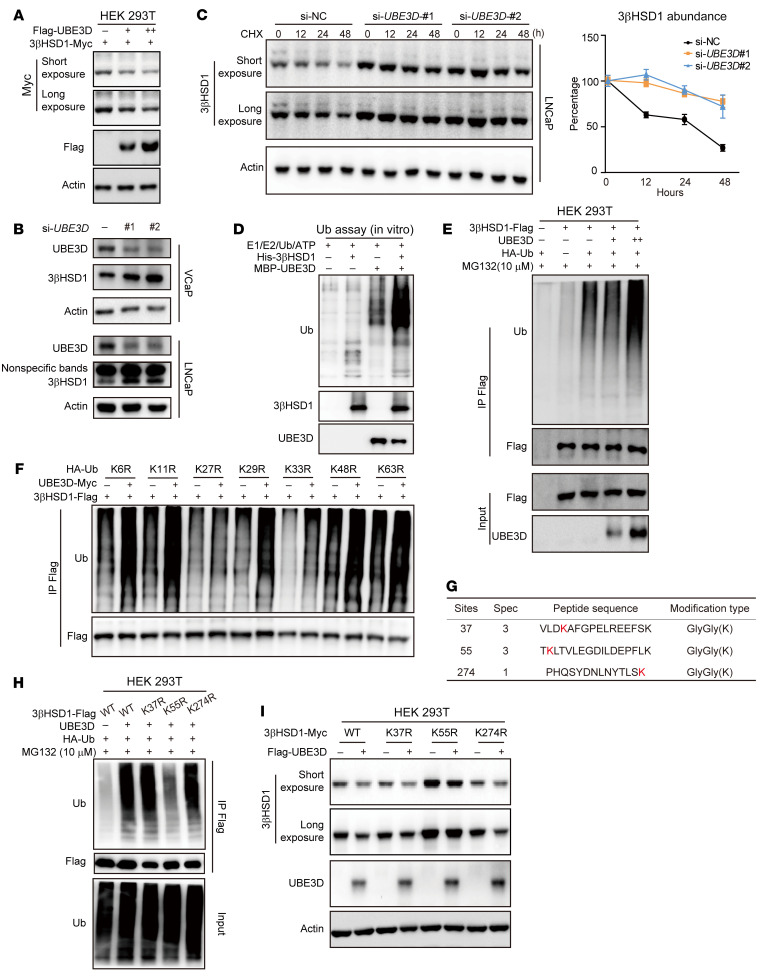
UBE3D ubiquitinates 3βHSD1. (**A**) 3βHSD1 levels in HEK293T cells overexpressing UBE3D. (**B**) Endogenous 3βHSD1 levels in LNCaP and VCaP cells with or without UBE3D knockdown. Different siRNAs were used to knock down endogenous UBE3D. (**C**) Half-life of endogenous 3βHSD1 in LNCaP cells with or without UBE3D knockdown. CHX, cycloheximide, 100 μM. (**D**) 3βHSD1 ubiquitylation in the reconstituted ubiquitylation bacterial system. (**E**) 3βHSD1 ubiquitylation in 293T cells with or without UBE3D overexpression. (**F**) K27-linked polyubiquitin (poly-Ub) chains were conjugated to 3βHSD1 by UBE3D. K27R, the K-to-R substitution only at K27 in Ub protein. (**G**) Potential 3βHSD1 ubiquitylation sites determined by mass spectrometry. The protein samples were obtained from an in vivo ubiquitination assay in HEK293T cells. (**H**) Identification of the ubiquitylation sites in 3βHSD1. K37R, K55R, and K274R: 3βHSD1-FLAG mutant with K-to-R substitutions at K37, K55, and K274, respectively. (**I**) Stabilities of different 3βHSD1 mutants in HEK293T cells with or without UBE3D overexpressed.

The regulatory mechanisms of UBE3D on 3βHSD1 were then further investigated. UBE3D overexpression reduced 3βHSD1 abundance in HEK293T cells, which was rescued by proteasome inhibitors (Figure 7A and Supplemental Figure 8A). UBE3D knockdown resulted in an accumulation of endogenous 3βHSD1 in LNCaP and VCaP cells (Figure 7B). UBE3D knockdown prolonged the half-life of 3βHSD1 in LNCaP and VCaP cells (Figure 7C and Supplemental Figure 8B). UBE3D enhanced 3βHSD1 ubiquitylation substantially in the reconstituted ubiquitylation system in vitro (Figure 7D). In 293T cells, UBE3D overexpression also promoted 3βHSD1 ubiquitylation in a dose-dependent manner, and HECT domain was essential for UBE3D-mediated 3βHSD1 ubiquitylation (Figure 7E and Supplemental Figure 8C). K27-linked ubiquitin chains were linked to 3βHSD1 by UBE3D (Figure 7F). The ubiquitylation sites on 3βHSD1 were further determined by IP-MS, and 3 potential sites (K37, K55, and K274) were identified (Figure 7G and Supplemental Figure 8D). Mutations on K55 diminished UBE3D-mediated 3βHSD1 ubiquitylation and abolished 3βHSD1 degradation, indicating that K55 is the main ubiquitylation site for UBE3D on 3βHSD1 (Figure 7, H and I, and Supplemental Figure 8E). The effect of UBE3D on different isoforms of 3βHSD1 was also determined. UBE3D degraded both 3βHSD1 (367T) and 3βHSD1 (367N) isoforms (Supplemental Figure 8F). Collectively, these results demonstrate that UBE3D ubiquitinates and degrades 3βHSD1.

**Figure 8:**
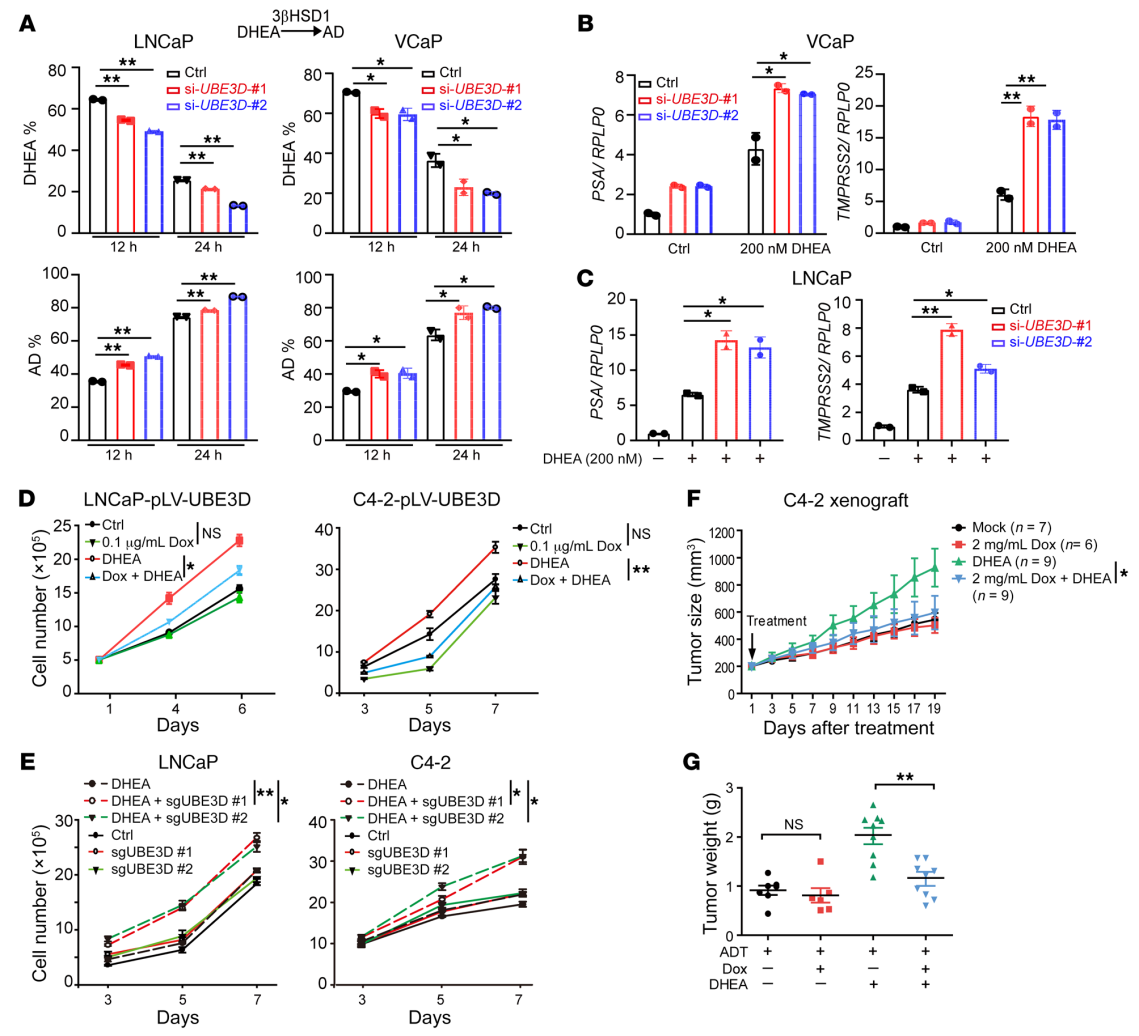
UBE3D deletion enhances tumor aggressiveness. (**A**) DHEA utilization in LNCaP and VCaP cells upon UBE3D knockdown. [^3^H]-DHEA was used to treat LNCaP and VCaP after UBE3D knockdown. (**B** and **C**) Expression of AR target genes in LNCaP and VCaP after UBE3D knockdown. Charcoal-stripped serum (CSS) was used for starvation before DHEA was added. (**D**) Cell proliferation in LNCaP and C4-2 with or without UBE3D overexpression. Prostate cancer cells with doxycycline-induced (Dox-induced) UBE3D overexpression were starved in CSS for 48 hours before DHEA or Dox treatment. (**E**) Cell proliferation in LNCaP and C4-2 with or without UBE3D knockout. Different guide RNAs were used to generate UBE3D-knockout cells in LNCaP and VCaP. (**F**) Effect of UBE3D on DHEA-induced xenograft growth. C4-2 cells with Dox-induced UBE3D overexpression were used for xenograft assay in castrated mice. DHEA treatment was achieved through sustained-release DHEA pellets. Dox, 2 mg/mL in water. (**G**) Tumor weights from xenograft assay. Results are shown as mean ± SD. *P < 0.05, **P < 0.01 by 1-way ANOVA.

To investigate the biological effects of UBE3D on tumor aggressiveness, endogenous UBE3D was knocked down in LNCaP and VCaP cells. The conversion of DHEA to AD was accelerated after UBE3D knockdown (Figure 8A). Consistently, the expression of AR target genes in LNCaP and VCaP cells was upregulated after UBE3D knockdown (Figure 8, B and C). Stable cell lines with UBE3D knockout or doxycycline-induced (Dox-induced) UBE3D overexpression were also generated in LNCaP and C4-2 cells (Supplemental Figure 9). UBE3D overexpression suppressed DHEA-induced cell proliferation markedly in both cell lines (Figure 8D). Stable cell lines with UBE3D knocked out grew faster after DHEA treatment (Figure 8E). The effect of UBE3D on DHEA-mediated tumor growth was then further tested in vivo. C4-2 cells with Dox-induced UBE3D expression were injected subcutaneously in castrated mice with or without sustained-release DHEA pellets implanted to mimic the endocrine environment of patients. The addition of Dox suppressed DHEA-induced growth substantially in mice (Figure 8F). Tumors with UBE3D overexpressed shrank as indicated by tumor weight (Figure 8G). Together, these data demonstrate that UBE3D regulates 3βHSD1 homeostasis and consequently affects DHEA utilization and tumor aggressiveness.

### Equilin as 3βHSD1 inhibitor for disease intervention.

**Figure 9:**
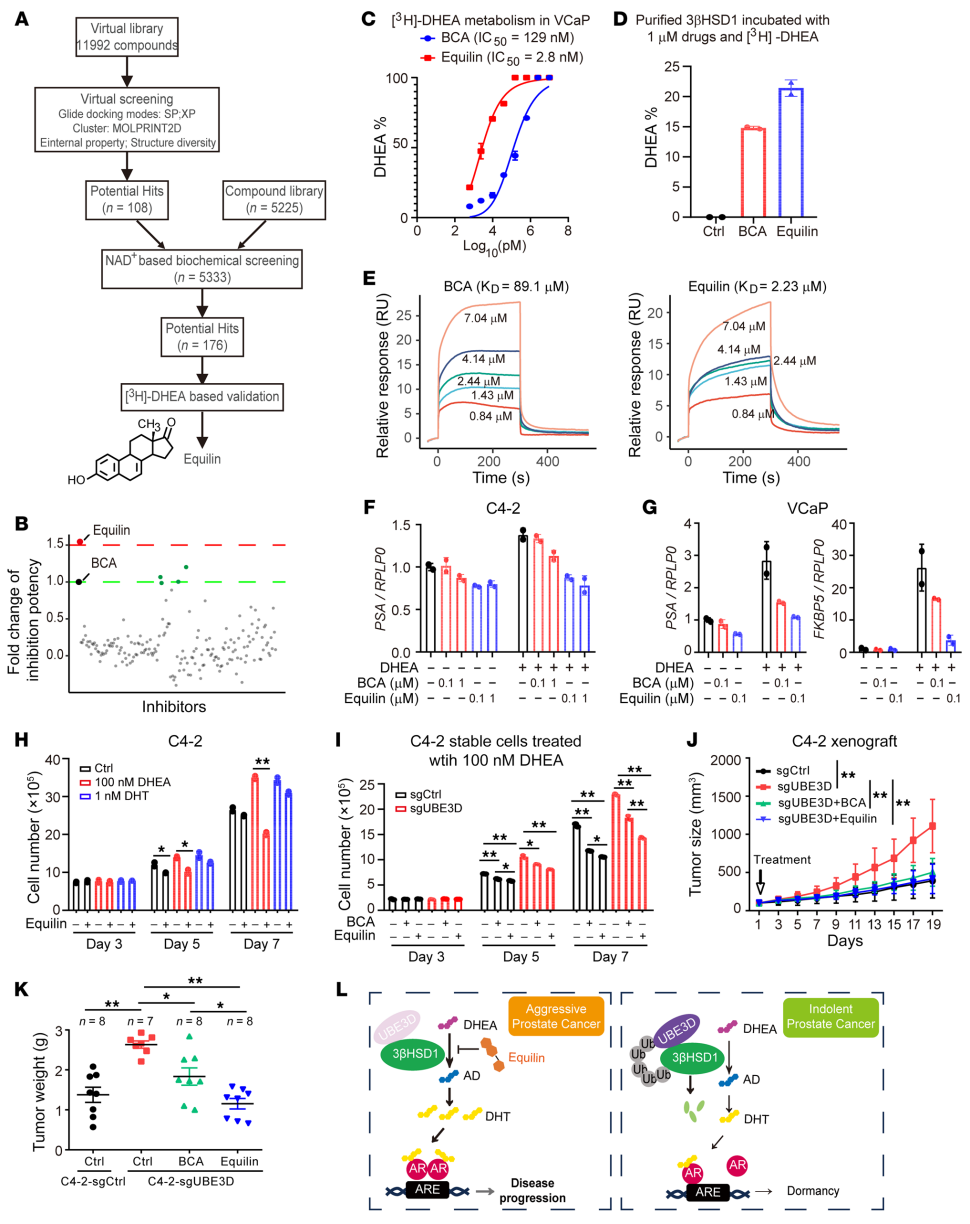
Equilin suppresses UBE3D-related tumor aggressiveness. (**A**) Schema of 3βHSD1 inhibitor screening. (**B**) Screening results of 176 potential hits. LNCaP cells were treated with [^3^H]-DHEA and potential hits for DHEA metabolism. Inhibitory efficacy was compared with that of biochanin A (BCA). (**C**) Equilin inhibited DHEA utilization in VCaP cells more potently. [^3^H]-DHEA was used to treat VCaP cells with different doses of equilin and BCA. (**D**) Equilin inhibited the activity of purified 3βHSD1 more potently. GST-3βHSD1 (2 μg) was used for the in vitro enzyme activity assays. (**E**) Affinity of equilin and BCA to purified 3βHSD1 protein determined by surface plasmon resonance technology. Data were fitted with a 1:1 kinetic binding model as binding affinity (*K_D_*) indicated. (**F** and **G**) Equilin inhibited the expression of AR target gene. DHEA, 100 nM. Charcoal-stripped serum was used for starvation before DHEA was added. (**H**) Effects of equilin on cell proliferation of C4-2 cells. Equilin, 2.5 μM. (**I**) Effects of equilin on cell proliferation of C4-2 cells with or without UBE3D knocked out. Equilin, 5 μM; BCA, 5 μM. One-way ANOVA. (**J**) Effects of equilin on xenograft growth. C4-2 cells with or without UBE3D knocked out were used for xenograft assay. Mice were castrated and implanted with sustained-release DHEA pellets. BCA, 50 mg/kg/d; equilin, 50 mg/kg/d. (**K**) Tumor weights from xenograft assay. (**L**) Schema for 3βHSD1 regulation in aggressive prostate cancer and related treatment with equilin. Results are shown as mean ± SD. *P < 0.05, **P < 0.01 by 2-tailed Student’s *t* test unless otherwise stated.

Inhibitors targeting 3βHSD1 are intrinsic solutions for DHEA metabolism–related tumor aggressiveness. Although several 3βHSD1 inhibitors have been identified, a systematic 3βHSD1 inhibitor screening platform has yet to be established (16, 17). A virtual screening platform was established with 3βHSD1 structure predicted by AlphaFold2 and optimized with previously reported 3βHSD1 inhibitors, including biochanin A (BCA) (34, 35). An NAD^+^-based biochemical screening platform with purified 3βHSD1 proteins was used for large-scale inhibitor screening. With more than 10,000 compounds for virtual screening and 5,225 extra compounds for biochemical screening, a total of 176 potential hits were identified (Figure 9A). These compounds were then used to treat LNCaP cells together with [^3^H]-DHEA for validation, and equilin was discovered as the most potent 3βHSD1 inhibitor (Figure 9B). Equilin inhibited 3βHSD1 activity better than BCA in different prostate cancer cells, with an IC_50_ around 2.8 nM in VCaP cells (Figure 9C and Supplemental Figure 10, A–C). Equilin also directly inhibited the activity of purified 3βHSD1 (Figure 9D). The direct binding of equilin to 3βHSD1 proteins was determined by surface plasmon resonance. Equilin showed a higher affinity to 3βHSD1 than BCA did (Figure 9E). The docking model with AlphaFold2-predicted 3βHSD1 structure also supports a higher affinity of equilin to 3βHSD1, compared with BCA (Supplemental Figure 11). Thus, equilin potently suppressed the expression of AR target genes in C4-2 and VCaP cells after DHEA treatment (Figure 9, F and G). Furthermore, equilin at a dose of 2.5 μM also suppressed DHEA-induced but not DHT-induced cell proliferation in C4-2 cells (Figure 9H). Knockout of endogenous UBE3D facilitated cell proliferation, and a higher dose of equilin (5 μM) was used to antagonize cell growth (Figure 9I). To determine whether 3βHSD1 is essential for the antitumor activity of equilin, C4-2 stable cell lines with 3βHSD1 knocked down were established. Equilin failed to suppress the expression of AR target genes and cell proliferation in 3βHSD1-depleted stable cell lines (Supplemental Figure 12). The antitumor activity of equilin was further tested in a mouse model. Xenografts with UBE3D knocked out showed increased aggressiveness and grew more rapidly. Equilin successfully antagonized tumor aggressiveness by suppressing tumor growth (Figure 9J). The inhibitory effect of equilin was also confirmed by tumor weights (Figure 9K). These data together demonstrate that equilin antagonizes tumor aggressiveness.

## Discussion

Limited efforts have been put into precision medicine for prostate cancer at early disease stages. Distinguishing patients at risk for aggressive prostate cancer before prostatectomy and ADT, or even before prostate cancer occurs, and developing related therapeutic, or even preventive, methods could fundamentally improve the clinical management of prostate cancer. Here we found that prostatic 3βHSD1 activity is essential for a deleterious androgenic environment to accelerate DHEA utilization and trigger the onset and early development of aggressive prostate cancer. UBE3D-mediated 3βHSD1 ubiquitylation was a clinically relevant mechanism for explaining patient heterogeneity of prostatic DHEA utilization. Equilin antagonizes aggressive prostate cancer as a 3βHSD1 inhibitor.

Considering the increased mutations as well as the fact that advanced prostate cancer is a highly heterogeneous disease, treatment resistance is inevitable, and clinical benefits are limited (36–38). Preventing the onset of aggressive prostate cancer or treating it at early stages could fundamentally change the field of prostate cancer treatment. Given that less than 20% of newly diagnosed prostate cancer will metastasize and become life-threatening, approaches for patient stratification and therapeutic targets for personalized treatment are essential to avoid overtreatment. Racial heterogeneity should also be taken into consideration in developing such strategies (39, 40). Although different genomic classifiers have been established to predict tumor aggressiveness, most of them aim for patients at late disease stages, or provide limited clues for novel therapeutic targets and strategies (41–44).

Androgens are major oncogenic metabolites for the initiation and early development of prostate cancer. DHT is synthesized in the prostate gland from testosterone originated from testis or DHEA from the adrenal gland. Circulating testosterone levels gradually decrease with age, while prostate cancer incidence increases with age. The activity of SRD5A2, which converts testosterone to DHT, declines with disease progression (9, 45). These facts indicate that testosterone is not the sole determinant of prostate cancer. It is yet undetermined how important adrenal DHEA is for prostate cancer progression at early disease stages. It is of interest only for humans and other primates, who have adrenal-secreted DHEA for DHT synthesis when abundant testosterone exists (46). The DHEA-to-DHT route provides more intermediate metabolites than the testosterone-to-DHT route. These intermediate metabolites are more stable, with detailed biological functions that have not been thoroughly investigated. Here we correlated prostatic DHEA metabolic features with tumor aggressiveness to find ways to distinguish patients with potential aggressive prostate cancer as early as possible. The DHEA-to-DHT route generates a polymorphic and heterogenic androgenic environment, which is conducive to aggressive prostate cancer onset and progression.

Dozens of enzymes participate in the conversion of DHEA to DHT, and it is difficult to comprehensively evaluate the activities of DHEA utilization in patients, making it impossible to investigate the physiological or pathological effects of DHEA utilization. Here, we treated fresh biopsies with [^3^H]-DHEA to evaluate prostatic DHEA utilization ex vivo and found that enhanced prostatic DHEA utilization is associated with tumor aggressiveness. With the aid of next-generation sequencing, we identified high-risk SNPs and gene mutations as well as gene signatures to reflect prostatic metabolic features, providing clinically feasible approaches for patient stratification. Both upstream regulators and downstream effectors of AR pathways are enriched in these signatures; thus it is not surprising to find the correlations of GWAS signature and transcriptomic signatures with steroidogenesis and AR pathways, respectively. Consistently, 3βHSD1 genotype has been proved to be a predictive biomarker for patient stratification (11, 12). However, because of the low frequency of *HSD3B1* *(1245C)* in East Asian populations, its application is limited to Western countries (18, 47). The GWAS signature and transcriptomic signature for DHEA utilization reflect prostatic 3βHSD1 activity and may serve as alternative approaches for patient stratification in different ethnicities. Risk alleles were enriched in patients with enhanced prostatic DHEA utilization, indicating a genetic background for patient heterogeneity and tumor occurrence. Higher mutation loads were observed in biopsies with suppressed DHEA utilization, indicating that androgen deprivation might increase tumor heterogeneity. Thus, androgens are not always a foe to patients, which explains the clinical benefits of bipolar androgen therapy and intermittent ADT (48–51). Also, *PTEN* deletion and other oncogenic gene mutations were frequently found in patients with low prostatic metabolic activity, indicating that multiple strategies are involved in disease progression. Prostatic DHEA utilization provides one but would not be the only aspect to identify at-risk patients.

Different mechanisms are involved in prostatic metabolic heterogeneity. UBE3D was found in our studies to regulate 3βHSD1 homeostasis, regardless of 3βHSD1 genotype, and negatively correlate with tumor aggressiveness in different databases. UBE3D is frequently deleted in advanced prostate cancer, and its expression is also varied substantially in patients at early disease stages. Patients with lower UBE3D abundance are at risk for the onset and early development of aggressive prostate cancer.

BCA has recently been reported as a potent 3βHSD1 inhibitor for suppressing prostate cancer progression, even after abiraterone and enzalutamide resistance (16, 17). However, a large-scale drug screening platform for 3βHSD1 has not yet been established. Here we optimized the virtual screening system and established a biochemical screening platform based on NAD^+^ detection. Equilin was discovered in our studies to be the most potent 3βHSD1 inhibitor after more than 17,000 compounds were screened. Equilin, also known as 7-dehydroestrone, is a natural estrogenic steroid synthesized in pregnant mares and used together with estrone and equilenin for hormone replacement therapy in postmenopausal woman to reduce coronary artery disease. Equilin modifies lipid profiles in patients and obtains neuroprotective and antioxidant activity (52–54). Here we found that equilin markedly suppresses the growth of UBE3D-deleted xenografts in mice as a 3βHSD1 inhibitor. With prostatic DHEA metabolic features as a predictive biomarker for aggressive prostate cancer, equilin might be potentially useful in the treatment of high-risk prostate cancer at early disease stages.

In conclusion, our results demonstrate the correlation between prostatic DHEA utilization and tumor aggressiveness at early disease stages. Genomic classifiers have been generated to evaluate prostatic DHEA utilization for patient stratification. UBE3D regulates 3βHSD1 homeostasis, and equilin antagonizes the development of tumor with potent 3βHSD1 activity (Figure 9L).

## Methods

### Preparation of human primary prostate tissue biopsies.

This investigation was conducted according to Declaration of Helsinki principles. A total of 524 primary prostatic biopsy samples were collected from 241 patients with increasing PSA or abnormal digital rectal exam results. The registered patients underwent transperineal ultrasound–guided systematic biopsy using an 18 G needle. Analysis of biochemical parameters including serum sex hormones (DHEA and AD) and prostate multiparametric MRI was performed before the procedure.

One-third of each biopsy sample (2.5 ± 1 mg) was used for metabolic profile analysis, one-third was used for whole-exome and transcriptome sequencing, and the remaining portion was fixed in formalin, paraffin-embedded, site-mounted, and assessed for pathology examination to determine the cancer cell content and Gleason score. Biopsy samples were washed with DMEM (Invitrogen), minced with razor blades, and then cultured in a 12-well plate at 37°C with DMEM (Invitrogen), 10% FBS (ExCell Bio), and penicillin-streptomycin (100×; Invitrogen) for immediate steroid metabolism assay.

### Steroidogenesis in patient biopsy samples.

Biopsy samples were treated with [^3^H]-labeled DHEA (100,000–200,000 cpm; final concentration 48 nM) (PerkinElmer). Two hundred fifty microliters medium was collected at 84 hours for HPLC analysis (35). Then samples were treated with β-glucuronidase (Novoprotein Scientific Inc.) at 37°C for 2 hours. Steroids were extracted with a mixture of ethyl acetate and isooctane (1:1), concentrated with a vacuum drier (Martin Christ Gefriertrocknungsanlagen), and resuspended with a mixture of methanol and water (1:1). An Acquity Arc System (Waters) and a β-RAM model 5 in-line radioactivity detector (LabLogic Systems) were used to analyze metabolites in samples. A mixture of [^3^H]-labeled androgens (AD, DHEA, progesterone, pregnenolone; PerkinElmer) was used as the standard to distinguish metabolites. The percentages of metabolites were calculated based on the area under the curve (AUC) for each metabolite. For example, DHEA % = (AUC of DHEA)/(AUC of DHEA + AUC of all DHEA metabolites) × 100%. Prostatic 3βHSD1 activity was calculated as: potent androgens %/(DHEA % + potent androgens %) × 100%. Based on prostatic 3βHSD1 activity, biopsies showing top 10% and bottom 10% activities with sufficient tissues for genomic and transcriptomic sequencing were selected for further analysis.

### Cell lines and materials.

LNCaP, C4-2, and HEK293T cells were purchased from the American Type Culture Collection and maintained in RPMI 1640 (LNCaP, C4-2) or DMEM (HEK293T) with 10% FBS (ExCell Bio). VCaP cells were provided by Jun Qin (Shanghai Institute of Nutrition and Health). All experiments with LNCaP and VCaP cells were done in plates coated with poly-dl-ornithine (Sigma-Aldrich). Cell lines were authenticated by Hybribio and determined to be mycoplasma free with primers 5′-GGGAGCAAACAGGATTAGATACCCT-3′ and 5′-TGCACCATCTGTCACTCTGTTAACCTC-3′. The following primary antibodies were used: anti-3βHSD1 (Abcam, ab55268), anti-UBE3D (Abcam, ab121927), anti-ubiquitin (Abways, CY5965), anti–glutathione *S*-transferase (anti-GST) (Cell Signaling Technology, 2624), anti-MYC (Millipore, 06-549), anti-FLAG (Sigma-Aldrich, H9658), and anti–β-actin (ABclonal, China, AC026). For stable cell lines, HEK293T cells were transfected with plvx-tight-puro-UBE3D, together with pMD2.G and pSAPX.2 plasmids. The supernatant was collected and used to infect LNCaP, VCaP, and C4-2 cells. Puromycin (1 μg/mL) and G418 (600 μg/mL) were used for selection. Doxycycline (Dox; 0.1 μg/mL; Sigma-Aldrich) was used to induce UBE3D expression. DHEA and DHT were purchased from Steraloids.

### RNA-Seq.

Total RNA from biopsy samples was extracted using AllPrep DNA/RNA/Protein Mini Kit (QIAGEN). VAHTS mRNA-seq V3 Library Prep Kit for Illumina (NR611) was used for library construction, following the manufacturer’s instructions. Briefly, 1,000 ng of total RNA was used for the purification and fragmentation of mRNA. Purified mRNA was subjected to first- and second-strand cDNA synthesis. cDNA was then ligated to sequencing adapters (VAHTS RNA Adapters set3–set6 for Illumina, N809/N810/N811/N812) and amplified by PCR (using 12 cycles). The final libraries were evaluated using a Qubit Fluorometer (Invitrogen) and QIAxcel Advanced System (QIAGEN). Next, sequencing was performed on NovaSeq 6000 (PE150, Illumina) by Berry Genomics Co. Ltd. The quality control of raw sequence data was evaluated by FastQC (v0.11.7; https://www.bioinformatics.babraham.ac.uk/projects/fastqc), and the quality trimming and adapter clipping were performed using Trimmomatic (v0.36-5; http://www.usadellab.org/cms/?page=trimmomatic). Paired-end reads were aligned to the GRCh38.91 human reference genome using HISAT2 (v2-2.1.0; http://daehwankimlab.github.io/hisat2/). Gene expression levels were quantified by HTSeq (v0.11.1; https://htseq.readthedocs.io/en/latest/). The normalization of counts was performed using DESeq2 (v1.24.0; https://bioconductor.org/packages/release/bioc/html/DESeq2.html). Differential expression analyses were performed using DESeq2 based on the gene read count data.

### DNA extraction and library construction.

Genomic DNA was extracted from patient prostatic biopsy samples using AllPrep DNA/RNA/Protein Mini Kit (QIAGEN). DNA quantification and integrity were determined by the Nanodrop spectrophotometer (Thermo Fisher Scientific) and 1% agarose electrophoresis, respectively. Genomic DNA samples were captured using Agilent SureSelect Human All Exon v6 library following the manufacturer’s protocol (Agilent Technologies). Briefly, approximately 130 μL (3 μg) genomic DNA was sheared to 150 to 220 bp small fragments using a sonicator (Covaris Inc.). The sheared DNA was purified and treated with reagents supplied with the kit according to the protocol. Adapters from Agilent were ligated onto the polished ends, and the libraries were amplified by PCR. The amplified libraries were hybridized with the custom probes. The DNA fragments bound with the probes were washed and eluted with the buffer provided in the kit. Then these libraries were sequenced on the Illumina sequencing platform (HiSeq X-10), and 150 bp paired-end reads were generated. The whole-exome sequencing and analysis were conducted by OE Biotech Co. Ltd.

### Preprocessing of sequencing reads.

The raw data were compiled in FASTQ format. In order to get high-quality reads that could be used for subsequent analysis, the raw reads were preprocessed with fastp (v0.19.5). Firstly, adapter sequences were trimmed. Bases in a sliding window with average quality value below 20 were also trimmed. Then reads with ambiguous bases or shorter than 75 bp were also removed. Clean reads were aligned to the reference human genome (GRCh37) using the Burrows-Wheeler Aligner (v0.7.12; https://github.com/lh3/bwa). The mapped reads were sorted and indexed using SAMtools (v1.4; https://github.com/samtools/samtools).

### Pathway enrichment and gene set enrichment analysis.

For pathway enrichment analysis, the differentially expressed genes were prepared for pathway enrichment with the MSigDB Investigate Gene Set module using hallmark gene sets (h.all.v7.2.symbols.gmt). For gene set enrichment analysis, normalized counts were prepared for analysis using GSEA 3.0. The hallmark gene sets (h.all.v7.2.symbols.gmt) were used, and the genes were ranked as Ratio_of_Classes or Signal2Noise. The permutation type selected was gene_set, and other sets followed the default set of GSEA. The thresholds for inclusion were *P* < 0.05 and *q* < 0.25. The GSEA plot, normalized enrichment score, and false discovery rate (FDR) *q* values were derived from GSEA output.

### Rare variants burden test.

To perform the gene-based burden test, high-confidence variants and variants that are likely pathogenic based on minor allele frequency were selected. Variants that met quality and pathogenicity filters are referred to as “qualifying variants.” For each gene, the number of individuals with high 3βHSD1 activity or low 3βHSD1 activity who carried at least 1 qualifying variant was counted. After tabulation of high 3βHSD1 activity group and low 3βHSD1 activity group counts, a 2 × 2 contingency table was generated for each gene. This contingency table represents the number of high 3βHSD1 activity group and low 3βHSD1 activity group members who carried and did not carry a qualifying variant in each gene. *P* values were calculated using 2-sided Fisher’s exact test.

### Variant calling.

The GATK HaplotypeCaller was used to conduct germline mutation calling (55). Mutect2 (https://gatk.broadinstitute.org/hc/en-us/articles/360037593851-Mutect2) was used to perform somatic SNV and indel calling in biopsy samples with matching blood sample. Many annotation databases, such as RefSeq, 1000 Genomes, the Catalogue of Somatic Mutations in Cancer (COSMIC), and OMIM, were referred to during SNP and indel calling and annotated using ANNOVAR (https://annovar.openbioinformatics.org/en/latest/). CNVkit was used to detect genomic segments with somatic copy number variations (CNVs) from whole-exome sequencing data from 54 tumors (56). In addition, 47 matched normal blood samples from this study were used to create a pooled reference to evaluate segment copy number, which was further used in processing all tumor samples. The GISTIC2 algorithm was used to detect recurrently amplified or deleted genomic regions with the following modified parameters: -ta, 0.1; -td, 0.1; -js, 4; -broad, 1; -brlen, 0.7; -conf, 0.99; -genegistic, 1; -savegene, 1 (57). The CNV level for all genes was extracted from the GISTIC output files (all_threshold_by_genes) using a cutoff of ±1.

To obtain the somatic indel and SNV frequency in patients, tier 1 genes of Cancer Gene Census in COSMIC were investigated, and the results of Mutect2 were converted to MAF file. A patient would be identified as a carrier when a mutation was detected in a gene (regardless of its alternative allele fraction) in any biopsy samples. The CNV frequency within a patient group was calculated as G-score output from GISTIC2, which reflects both the degree of an alteration and its frequency in the group.

### Latent variable hierarchical model.

The hierarchical model consists of 3 layers, with a set of genes as the bottom layer representing the transcriptomic signature underlying 3βHSD1 activity. 3βHSD1 activity was modeled as a hidden variable *Z* at the second layer. 3βHSD1 activity consequently influences disease-free survival of prostate cancer, which is modeled as the final observable layer. Random forest algorithm was used to determine genes associated with *Z* (3βHSD1 activity), and disease-free survival analysis based on biochemical recurrence with TCGA database was further integrated to parameterize this hidden variable model (58).

### Performance assessment of gene signature.

Biopsy samples were split into 2 groups, according to the ratio of high 3βHSD1 activity group to low 3βHSD1 activity group, with one group as training set and the other as test set. Random sampling of 200 times was performed to evaluate the performance of the signature and individual gene with random forest algorithm. The predictive ability of the signature and individual gene for 3βHSD1 activity was compared using area under the receiver operating characteristic curve (AUC). The prognostic ability of the signature and individual gene was compared using hazard ratios and *P* values of survival analysis.

### 3βHSD1 activity–related polygenic risk score calculation.

Polygenic risk scores (PRSs) were generated using sets of prostate cancer–associated variants in UKBB and PRACTICAL (24). To obtain the optimal set of PRS variants, the *P* value threshold for GWAS resulting in the best-performing PRS was defined based on the maximum amount of variants required for 3βHSD1 activity and PRS to achieve a significant association. Variants with a *P* value below the threshold were selected as the optimal-variants set.

Standard approaches were used to generate a PRS for each individual (59). For each PRS variant, risk alleles were counted for each individual, i.e., the allele dosage at locus *i* in individual *j* (*d_i,j_*) ranges from 0 to 2. Mean counts of risk alleles for each study site were used to fill in any missing genotype data. This was done to avoid biases whereby individuals with more missing data have lower polygenic scores. For each risk score, allele dosages were weighted using GWAS effect sizes *β*. PRSs were generated for each individual by summing across L loci:

 Equation 1

### Survival analysis.

Log-rank test was used for Kaplan-Meier survival curves. Cox proportional-hazards regression models were used to generate hazard ratios and 95% confidence intervals.

### Gene expression assay.

Cells were starved for at least 48 hours with phenol red–free and 10% charcoal-stripped serum (Lonsera) and treated with DHEA (Steraloids) for 24 hours. Cell to cDNA Kit (EZBioscience) was used for cDNA synthesis directly from cells. Quantitative PCR (qPCR) experiment was conducted with a Bio-Rad CFX96 using EZBioscience 2′ SYBR Green qPCR master mix. The primers for qPCR have been described in a previous study (20). Results are presented as the mean and SD value from 1 representative experiment. All gene expression assays were performed in technical duplication and repeated at least 3 times in independent experiments.

### Protein purification and GST binding assay.

DNA fragment corresponding to UBE3D was cloned into the pMAL-C2X-3C vector. The MBP-UBE3D proteins were expressed in the BL21 (DE3) strain. Then the bacteria were grown to an optical density (OD_600_) of about 0.6, with 0.3 mM IPTG to induce protein expression at 16°C for 16 hours. Soluble UBE3D was enriched with MBP Resin (Biolab), and 10 mM maltose was used for elution. Hitrap Q anion exchange column (GE Healthcare) and HiLoad 26/60 Superdex 200 gel filtration column (GE Healthcare) were used for further purification.

The human 3βHSD1 gene (accession NP_000853.1) was codon optimized and subcloned into a pFastBac vector (Invitrogen) with amino-terminal 10× His tag. Baculovirus was generated with the Bac-to-Bac system (Invitrogen) and used for infecting *Spodoptera frugiperda* (Sf9) cells (Union-Bio, China) at a density of 2 × 10^6^ cells per mL and 10 mL of virus per liter of cells. Infected cells were collected after 60 hours by centrifugation, frozen in liquid nitrogen, and stored at −80°C. Cell pellets were disrupted using a Dounce tissue grinder (Sigma-Aldrich) on ice with lysis buffer (25 mM HEPES [pH 7.5], 150 mM NaCl, 5% vol/vol glycerol) and then solubilized with 0.08% (wt/vol) Triton X-100 (Sigma-Aldrich) at 4°C for 2 hours. After centrifugation (55,000*g*, 45 minutes, 4°C), 3βHSD1 was purified from the supernatant using a Ni^2+^-nitrilotriacetate affinity resin (QIAGEN), 0.5 mL resin per liter cell culture. 3βHSD1 was then concentrated to around 5 mg/mL (Amicon, 50 kDa cutoff; Millipore) and loaded onto a Superdex 200 Increase 10/300 GL size-exclusion column (Thermo Fisher Scientific) equilibrated with 25 mM HEPES (pH 7.5), 150 mM NaCl, and 0.03% Triton X-100. Purified 3βHSD1 was concentrated to around 10 mg/mL for the assay.

GST and GST-3βHSD1 proteins were respectively mixed with glutathione resin (Sigma-Aldrich, G4510) in PBS with cocktail and incubated overnight. MBP-UBE3D was added into the mixture and incubated for 2 hours at 4°C. Then beads were washed extensively with NP-40 lysis buffer (50 mM Tris-HCl [pH 7.6], 150 mM NaCl, 5 mM EDTA, 1% NP-40, and 1.0% protease inhibitor cocktail [Roche]). Proteins bound to glutathione beads were boiled at 100°C for 10 minutes, and detected by immunoblotting with indicated antibodies.

### In vitro ubiquitination assay.

The experiment was performed using an in vitro ubiquitination assay kit (Enzo Life Sciences, BML-UW9920-0001) according to the manufacturer’s instructions. 3βHSD1 protein was added into each reaction buffer containing 100 nM E1 ligase (Boston Biochem), 0.5 mM E2 ligase, and 1× Ubiquitin Conjugation Reaction Buffer containing ATP in the presence or absence of 100 ng UBE3D proteins. Reactions were allowed to proceed at 37°C for 1 hour.

### Mouse xenograft studies.

Male B-NDG [B: Biocytogen; N: NOD background; D: DNAPK (Prkdc) null; G: IL2rgknockout] mice (aged 4–6 weeks) were obtained from Beijing Biocytogen. Cells (1 × 10^7^) were implanted subcutaneously into the right flank of the intact mice with Matrigel (Corning, 354234). Mice were castrated, implanted with DHEA sustained-release pellets (EZBioscience, China), and randomly assigned into different groups when the xenografts reached approximately 200 mm^3^ (length × width × width × 0.5). Stratified randomization was applied. Mice were first separated into different groups according to the tumor size. Sucrose control (5% sucrose) and doxycycline (2 mg/mL and 5% sucrose in water) containing water were replaced every 2 days. Equilin and biochanin A (BCA), both at a dose of 50 mg/kg/d, were used for equilin function assay. Tumor growth was measured every 2 days with a caliper. Two-tailed Student’s *t* test was used for significance calculation; *P < 0.05, **P < 0.01.

### Mass spectrometry for detection of ubiquitination sites.

UBE3D was overexpressed in HEK293T cells and enriched with agarose beads. TCEP reduction, NEM alkylation, and trypsin digestion were performed sequentially. Peptides were separated by the EASY-nLC system (Thermo Fisher Scientific) and analyzed using a Q Exactive mass spectrometer (Thermo Fisher Scientific). Protein and ubiquitylation analysis was performed with Thermo Proteome Discoverer 2.1 (Thermo Fisher Scientific), and identified proteins were searched against the UniProt Human database (http://www.uniprot.org/).

### Virtual screening and molecular docking.

The structural model of human 3βHSD1 was built by AlphaFold and combined with the NAD^+^ cofactor and substrate DHEA. Then protein-ligand complex minimization was carried out by Prime (https://www.schrodinger.com/products/prime), and the minimized complex was used as the receptor for virtual screening. The structures of the ligands from the small-molecule library were prepared by LigPrep and screened by Glide at SP and XP precision in Schrödinger software. The compounds for bioassay validation were selected by a comprehensive consideration of the docking score, docked poses, structure diversity, and other factors. The 3D structure of the docked protein-ligand complex and the 2D protein-ligand interaction diagrams were presented by Maestro (https://www.schrodinger.com/products/maestro).

### NAD^+^-based biochemical screening.

The biochemical screening experiment was performed using a NAD(P)H-Glo Detection System Kit (Promega, G9061) in 384-well plates according to the manufacturer’s instructions. Firstly, purified 3βHSD1 protein solution was added into 1× PBS Buffer (Abcone, P41970) containing 0.1% BSA (Thermo Fisher Scientific, 37525) and 10 μM inhibitors from the small-molecule library. The reaction was incubated for 30 minutes at 4°C. Secondly, 1 mM NAD^+^ and 1 μM DHEA were added into each reaction sample, and the reaction was allowed to proceed at 37°C for 15 minutes. Lastly, the detection of NADH production was initiated by addition of an equal volume of NAD(P)H-Glo Detection Reagent, which contains reductase, reductase substrate, and luciferin detection reagent, to the reaction sample. Then luminescence was measured with an Envision instrument (PerkinElmer), and the available compounds were identified.

### Surface plasmon resonance binding assays.

The purified 3βHSD1 protein solution was first diluted with 10 mM sodium acetate (pH 5.5) to the concentration of 200 μg/mL. Then the protein was injected at a constant flow rate of 10 μL/min for 900 seconds and immobilized to CM7 sensor chips (GE Healthcare, UK) using the amine coupling kit. All surface plasmon resonance measurements were performed at 25°C using a Biacore 8K instrument (GE Healthcare, UK) in running buffer containing 1× PBS Buffer (pH 7.4) and 1% DMSO at a flow rate of 30 μL/min. To determine the binding affinities, BCA and equilin were analyzed using concentration response experiments. The resulting increasing concentrations of the analytes were injected over purified 3βHSD1 protein in the running buffer for 300 seconds. The surface was washed between each binding cycle with running buffer for 240 seconds, and the analyte was fully dissociated. Both experiments were analyzed by fitting with a 1:1 kinetic binding model to determine the *K_D_*, *K_a_*, and *K_d_* values of different analytes in the Biacore 8K evaluation software.

### In vitro 3βHSD1 activity assay.

3βHSD1 protein activity was measured by HPLC technology in vitro. Purified 3βHSD1 protein solution was added into 1× PBS Buffer (Abcone, P41970) containing 200 μM NAD^+^, 1 μM inhibitors, [^3^H]-labeled DHEA, and 0.1% BSA. Reactions were allowed to proceed at 37°C for 30 minutes.

### Statistics.

Unpaired 1-tailed and 2-tailed Student’s *t* test, 1-way and 2-way ANOVA, and log-rank test were performed to compare the differences between groups. FDR correction was used for multiple Student’s *t* test, and Tukey’s correction was used for ANOVA. Cox proportional-hazards regression models were used to generate hazard ratios and 95% confidence intervals. Pearson’s correlation coefficient was used for the correlation analysis. *P* values of less than 0.05 were considered significant. All analyses were performed using R (https://www.R-project.org/) 3.6.3 software. Data represent the median ± interquartile range unless indicated otherwise.

### Study approval.

All mouse experiments were approved by the Institutional Animal Care and Use Committee of the Center for Excellence in Molecular Cell Science. Collection of the biopsy samples was performed according to the relevant ethical standards and was approved by the Ethics Committee of Tongji Hospital. All registered patients provided informed consent.

### Data availability.

All sequencing data generated during this study were deposited in the National Omics Data Encyclopedia (https://www.biosino.org/node; accession OEP 003830). Data included in this article are provided in the Supporting Data Values file and are also available upon request from the authors.

## Author contributions

Z Li, DW, and XL designed the study. XZ, SH, DH, QZ, TY, QT, Z Liu, WY, RR, CW, JL, HT, and YL performed the experiments. Z Li, Zengming Wang and Zixian Wang analyzed the data. Z Li, XL, WB, and GW wrote the manuscript. All authors discussed the results and comments on the manuscript.

## Supplementary Material

Supplemental data

Supporting data values

## Version 1 12/15/2023

Electronic publication
