# Supplementary material for: Active DHEA uptake in the prostate gland correlates with aggressive prostate cancer
Source: J Clin Invest. 2023 Dec 15;133(24):e171199. doi: 10.1172/JCI171199 (PMC10721157; doi:10.1172/JCI171199)
Supplement: Supplemental data [file jci-133-171199-s228.pdf]

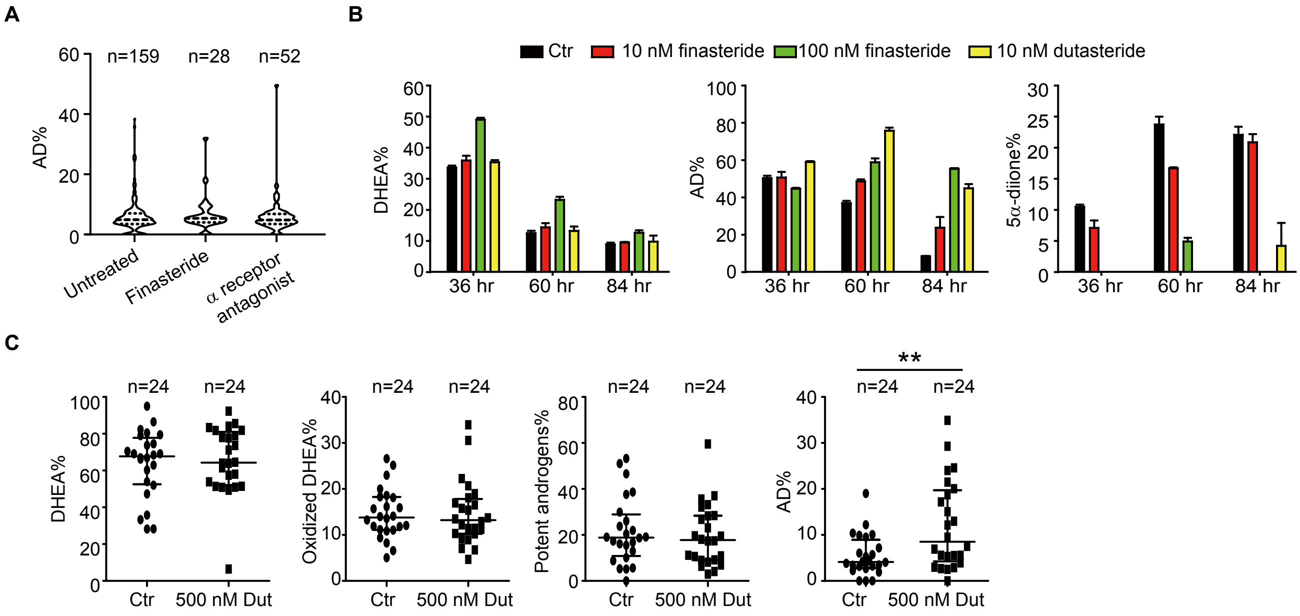
A
B
C
AD%
n=159
n=28
n=52
Untreated
Finasteride
α receptor antagonist
Ctr
10 nM finasteride
100 nM finasteride
10 nM dutasteride
DHEA%
5α-dione%
36 hr
60 hr
84 hr
n=24
Oxidized DHEA%
Potent androgens%
**
Ctr
500 nM Dut

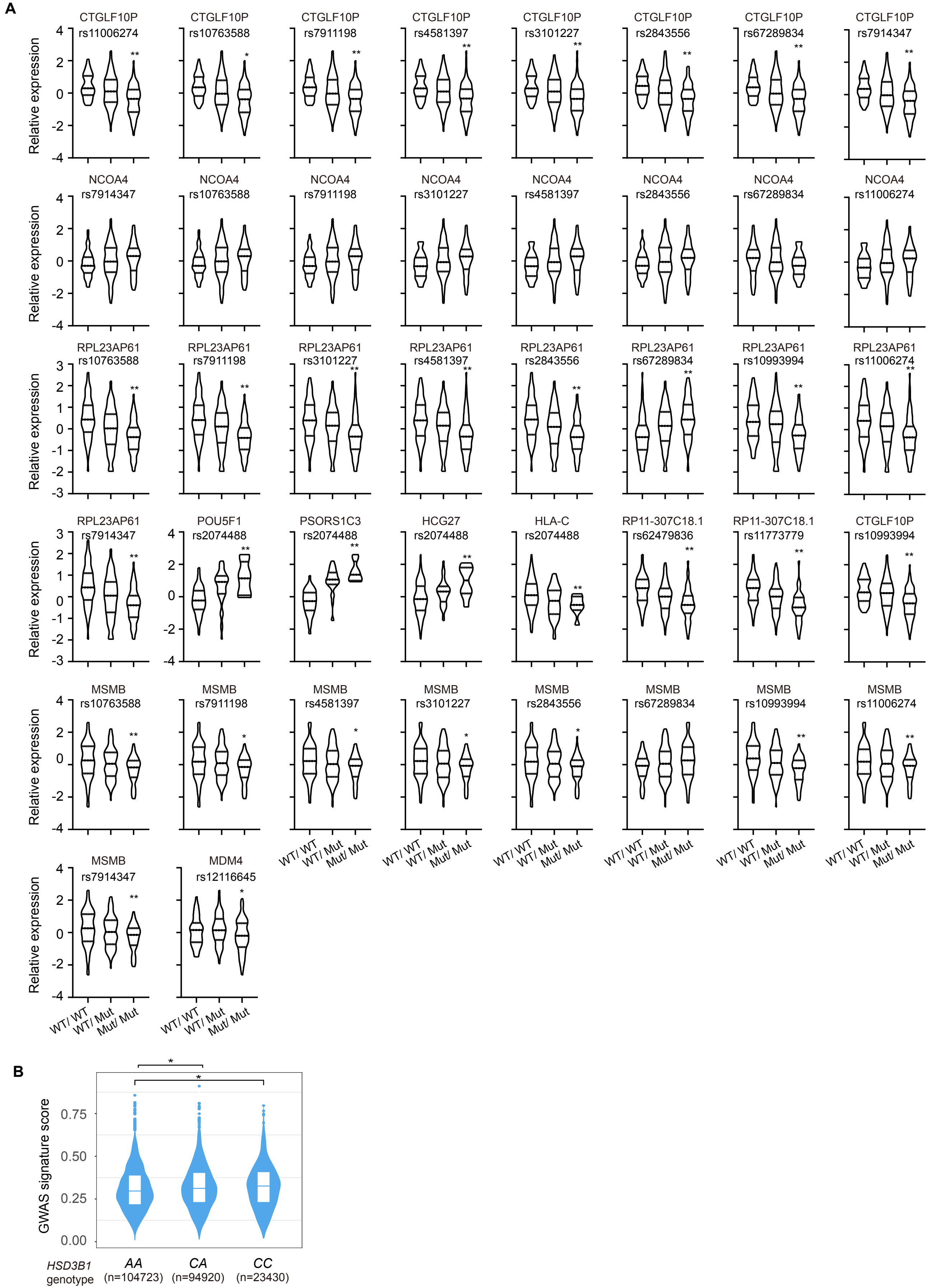
A
Relative expression
CTGLF10P rs11006274
CTGLF10P rs10763588
CTGLF10P rs7911198
CTGLF10P rs4581397
CTGLF10P rs3101227
CTGLF10P rs2843556
CTGLF10P rs67289834
CTGLF10P rs7914347
NCOA4 rs7914347
NCOA4 rs10763588
NCOA4 rs7911198
NCOA4 rs3101227
NCOA4 rs4581397
NCOA4 rs2843556
NCOA4 rs67289834
NCOA4 rs11006274
RPL23AP61 rs10763588
RPL23AP61 rs7911198
RPL23AP61 rs3101227
RPL23AP61 rs4581397
RPL23AP61 rs2843556
RPL23AP61 rs67289834
RPL23AP61 rs10993994
RPL23AP61 rs11006274
RPL23AP61 rs7914347
POU5F1 rs2074488
PSORS1C3 rs2074488
HCG27 rs2074488
HLA-C rs2074488
RP11-307C18.1 rs62479836
RP11-307C18.1 rs11773779
CTGLF10P rs10993994
MSMB rs10763588
MSMB rs7911198
MSMB rs4581397
MSMB rs3101227
MSMB rs2843556
MSMB rs67289834
MSMB rs10993994
MSMB rs11006274
MSMB rs7914347
MDM4 rs12116645
WT/ WT
WT/ Mut
Mut/ Mut
B
GWAS signature score
0.00
0.25
0.50
0.75
HSD3B1 genotype
AA (n=104723)
CA (n=94920)
CC (n=23430)

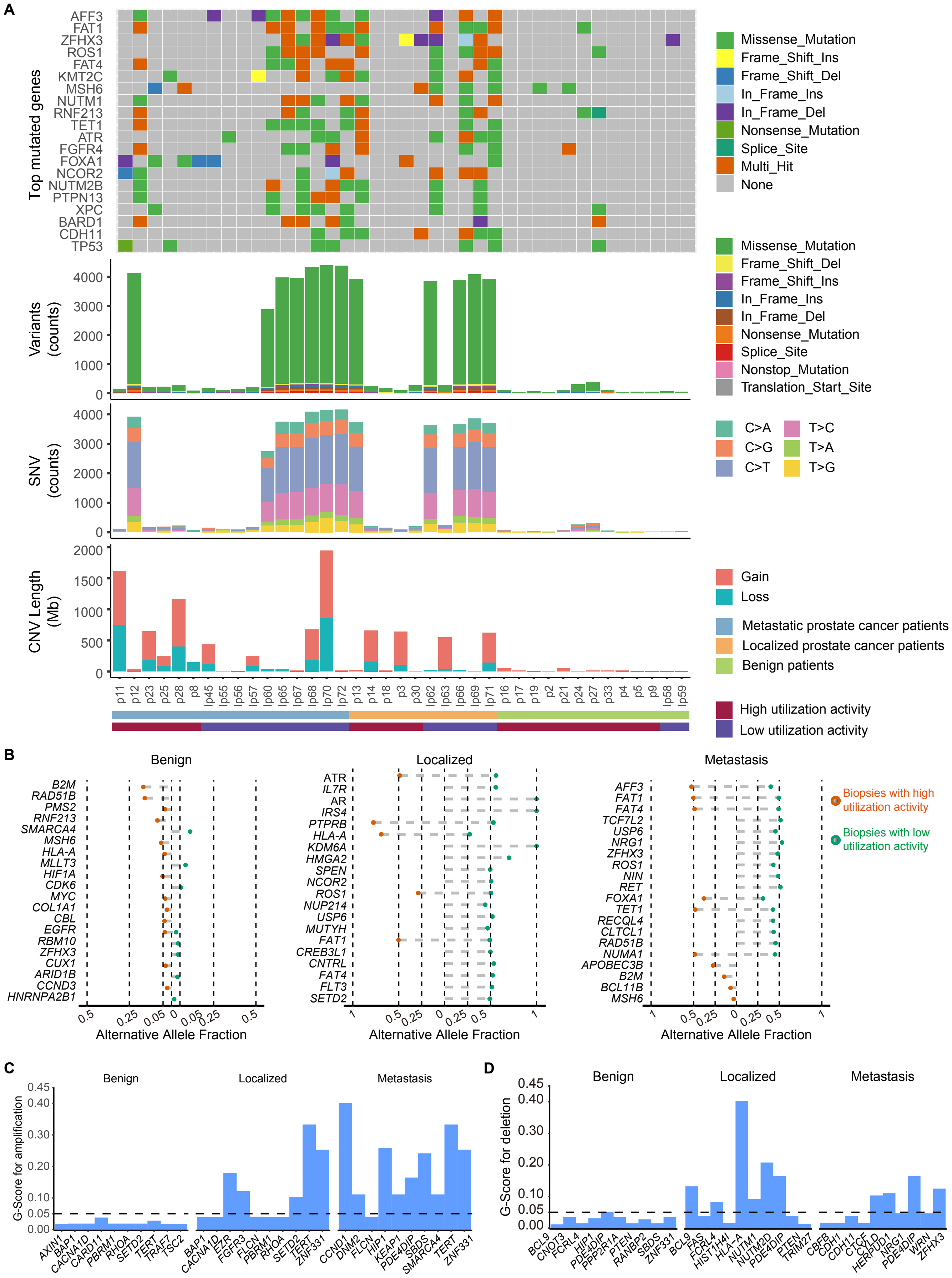
A
Top mutated genes
AFF3
FAT1
ZFHX3
ROS1
FAT4
KMT2C
MSH6
NUTM1
RNF213
TET1
ATR
FGFR4
FOXA1
NCOR2
NUTM2B
PTPN13
XPC
BARD1
CDH11
TP53
Missense_Mutation
Frame_Shift_Ins
Frame_Shift_Del
In_Frame_Ins
In_Frame_Del
Nonsense_Mutation
Splice_Site
Multi_Hit
None
Variants (counts)
Missense_Mutation
Frame_Shift_Del
Frame_Shift_Ins
In_Frame_Ins
In_Frame_Del
Nonsense_Mutation
Splice_Site
Nonstop_Mutation
Translation_Start_Site
SNV (counts)
C>A
C>G
C>T
T>C
T>A
T>G
CNV Length (Mb)
Gain
Loss
Metastatic prostate cancer patients
Localized prostate cancer patients
Benign patients
p11 p12 p23 p25 p28 p8 lp45 lp55 lp56 lp57 lp60 lp65 lp67 lp68 lp70 lp72 p13 p14 p18 p3 p30 lp62 lp63 lp66 lp69 lp71 p16 p17 p19 p2 p21 p24 p27 p33 p4 p5 p9 lp58 lp59
High utilization activity
Low utilization activity
B
Benign
B2M
RAD51B
PMS2
RNF213
SMARCA4
MSH6
HLA-A
MLLT3
HIF1A
CDK6
MYC
COL1A1
CBL
EGFR
RBM10
ZFHX3
CUX1
ARID1B
CCND3
HNRNPA2B1
Alternative Allele Fraction
Localized
ATR
IL7R
AR
IRS4
PTPRB
HLA-A
KDM6A
HMGA2
SPEN
NCOR2
ROS1
NUP214
USP6
MUTYH
FAT1
CREB3L1
CNTRL
FAT4
FLT3
SETD2
Alternative Allele Fraction
Metastasis
AFF3
FAT1
FAT4
TCF7L2
USP6
NRG1
ZFHX3
ROS1
NIN
RET
FOXA1
TET1
RECQL4
CLTCL1
RAD51B
NUMA1
APOBEC3B
B2M
BCL11B
MSH6
Alternative Allele Fraction
Biopsies with high utilization activity
Biopsies with low utilization activity
C
G-Score for amplification
Benign
AXIN1 BAP1 CACNA1D CARD11 PBRM1 RHOA SETD2 TERT TRAF7 TSC2
Localized
BAP1 CACNA1D EZR FGFR3 FLCN PBRM1 RHOA SETD2 TERT ZNF331
Metastasis
CCND1 DNM2 FLCN HIP1 KEAP1 PDE4DIP SBDS SMARCA4 TERT ZNF331
D
G-Score for deletion
Benign
BCL9 CNOT3 FCRL4 HIP1 PDE4DIP PPP2R1A PTEN RANBP2 SBDS ZNF331
Localized
BCL9 FAS FCRL4 HIST1H4I HLA-A NUTM1 NUTM2D PDE4DIP PTEN TRIM27
Metastasis
CBFB CDH11 CTCF CYLD HERPUD1 NRG1 PDE4DIP WRN ZFHX3

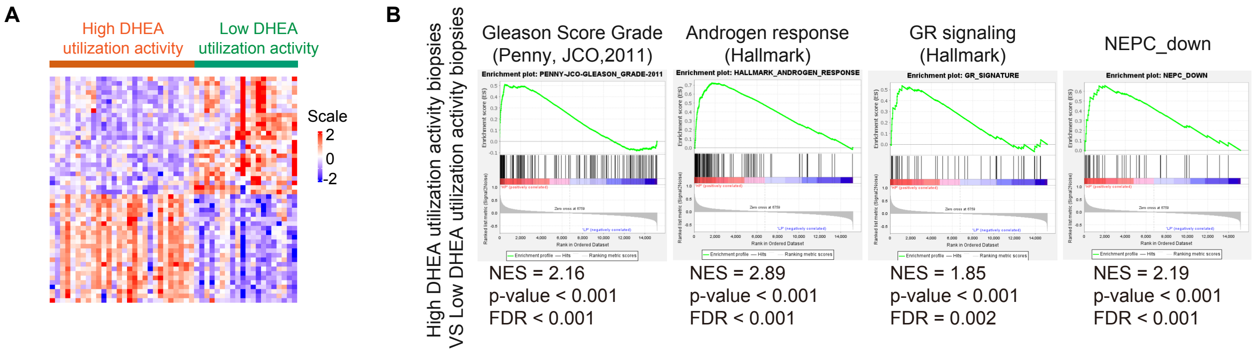
A
High DHEA utilization activity
Low DHEA utilization activity
Scale
2
0
-2
B
High DHEA utilization activity biopsies VS Low DHEA utilization activity biopsies
Gleason Score Grade (Penny, JCO,2011)
Androgen response (Hallmark)
GR signaling (Hallmark)
NEPC_down
NES = 2.16
p-value < 0.001
FDR < 0.001
NES = 2.89
p-value < 0.001
FDR < 0.001
NES = 1.85
p-value < 0.001
FDR = 0.002
NES = 2.19
p-value < 0.001
FDR < 0.001

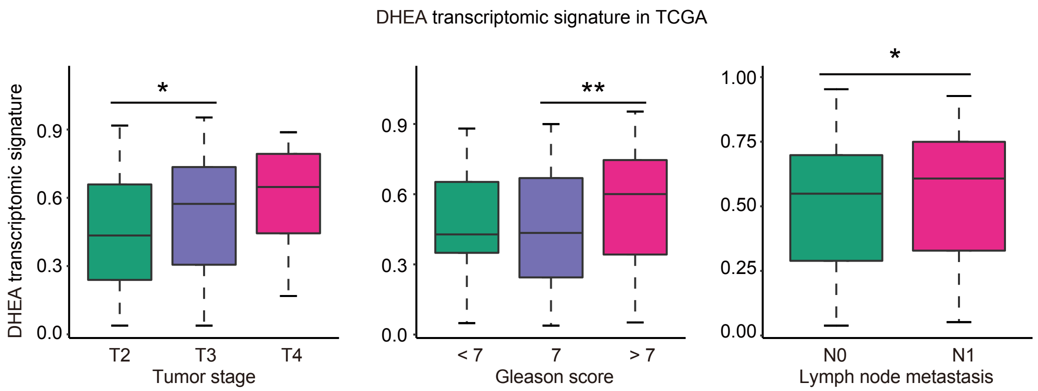
DHEA transcriptomic signature in TCGA
DHEA transcriptomic signature
0.0
0.3
0.6
0.9
*
T2
T3
T4
Tumor stage
0.0
0.3
0.6
0.9
**
< 7
7
> 7
Gleason score
0.00
0.25
0.50
0.75
1.00
*
N0
N1
Lymph node metastasis

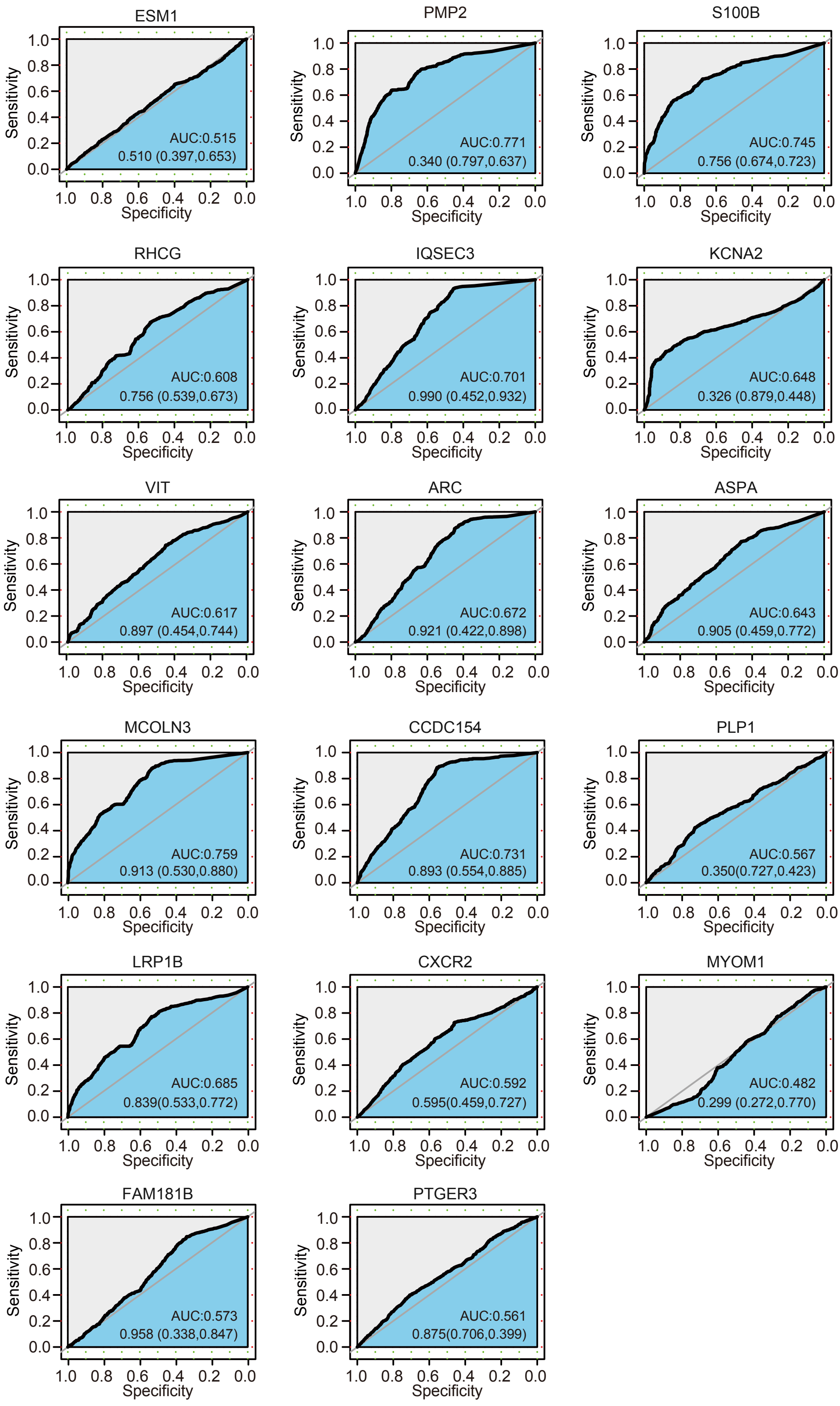

ESM1
AUC:0.515
0.510 (0.397,0.653)
PMP2
AUC:0.771
0.340 (0.797,0.637)
S100B
AUC:0.745
0.756 (0.674,0.723)
RHCG
AUC:0.608
0.756 (0.539,0.673)
IQSEC3
AUC:0.701
0.990 (0.452,0.932)
KCNA2
AUC:0.648
0.326 (0.879,0.448)
VIT
AUC:0.617
0.897 (0.454,0.744)
ARC
AUC:0.672
0.921 (0.422,0.898)
ASPA
AUC:0.643
0.905 (0.459,0.772)
MCOLN3
AUC:0.759
0.913 (0.530,0.880)
CCDC154
AUC:0.731
0.893 (0.554,0.885)
PLP1
AUC:0.567
0.350(0.727,0.423)
LRP1B
AUC:0.685
0.839(0.533,0.772)
CXCR2
AUC:0.592
0.595(0.459,0.727)
MYOM1
AUC:0.482
0.299 (0.272,0.770)
FAM181B
AUC:0.573
0.958 (0.338,0.847)
PTGER3
AUC:0.561
0.875(0.706,0.399)
Sensitivity
Specificity

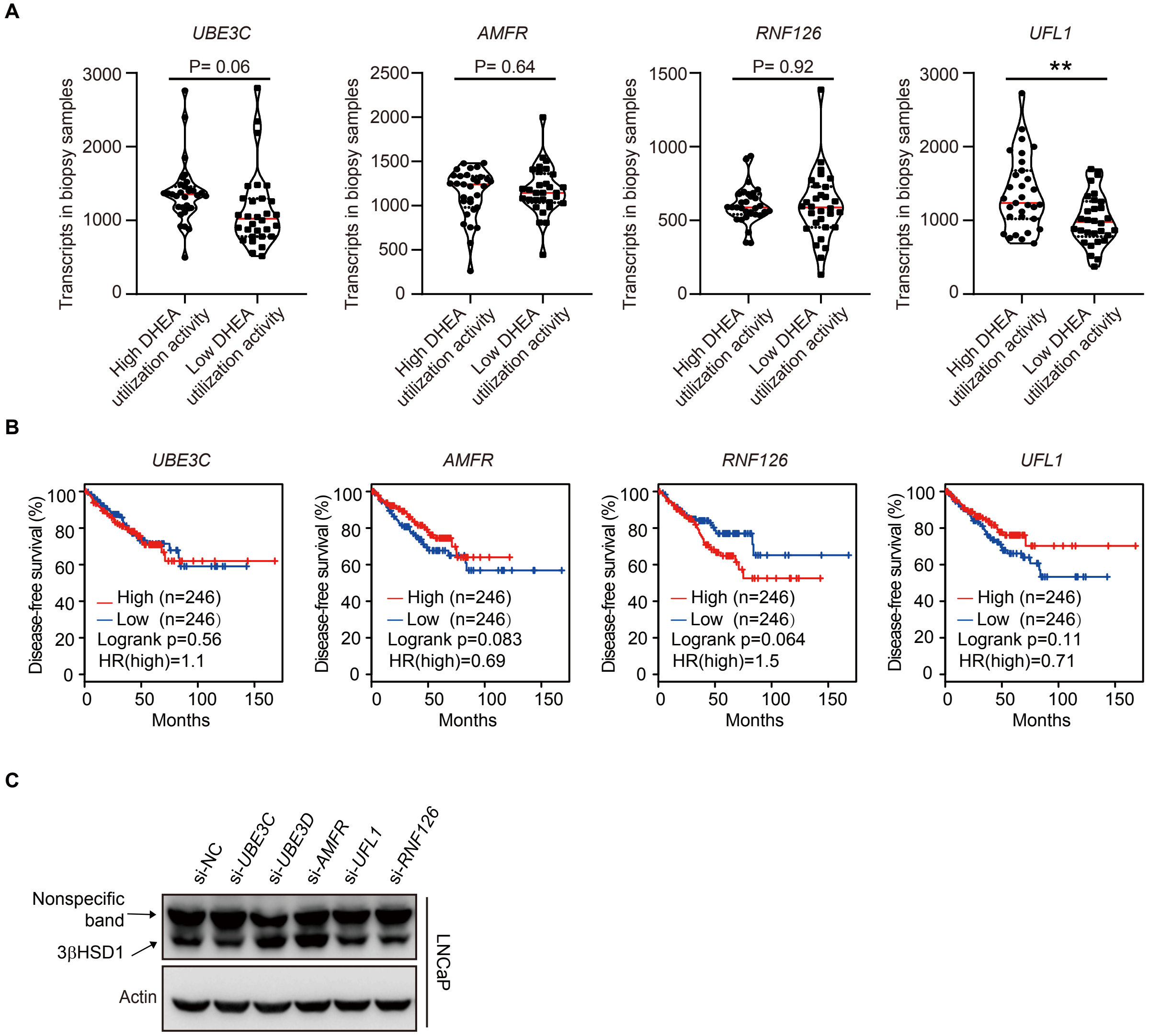
A
UBE3C
AMFR
RNF126
UFL1
P= 0.06
P= 0.64
P= 0.92
**
Transcripts in biopsy samples
High DHEA utilization activity
Low DHEA utilization activity
B
Disease-free survival (%)
Months
High (n=246)
Low (n=246)
Logrank p=0.56
HR(high)=1.1
Logrank p=0.083
HR(high)=0.69
Logrank p=0.064
HR(high)=1.5
Logrank p=0.11
HR(high)=0.71
C
si-NC
si-UBE3C
si-UBE3D
si-AMFR
si-UFL1
si-RNF126
Nonspecific band
3βHSD1
Actin
LNCaP

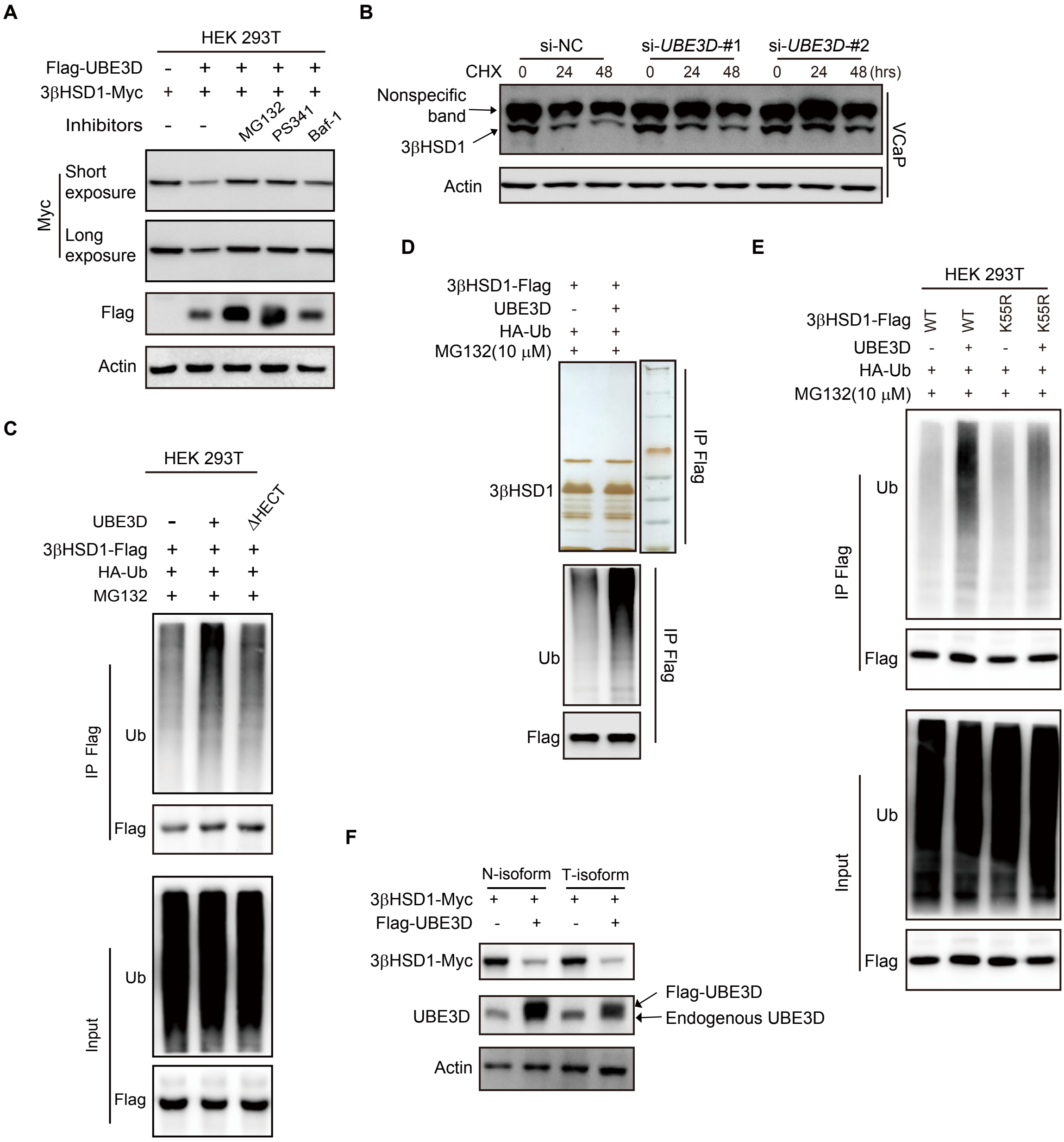
A
HEK 293T
Flag-UBE3D
3βHSD1-Myc
Inhibitors
MG132
PS341
Baf-1
Myc
Short exposure
Long exposure
Flag
Actin
B
si-NC
si-UBE3D-#1
si-UBE3D-#2
CHX
0
24
48
(hrs)
Nonspecific band
3βHSD1
Actin
VCaP
C
HEK 293T
UBE3D
ΔHECT
3βHSD1-Flag
HA-Ub
MG132
IP Flag
Ub
Flag
Input
Ub
Flag
D
3βHSD1-Flag
UBE3D
HA-Ub
MG132(10 μM)
3βHSD1
IP Flag
Ub
Flag
IP Flag
E
HEK 293T
3βHSD1-Flag
WT
WT
K55R
K55R
UBE3D
HA-Ub
MG132(10 μM)
IP Flag
Ub
Flag
Input
Ub
Flag
F
N-isoform
T-isoform
3βHSD1-Myc
Flag-UBE3D
3βHSD1-Myc
UBE3D
Flag-UBE3D
Endogenous UBE3D
Actin

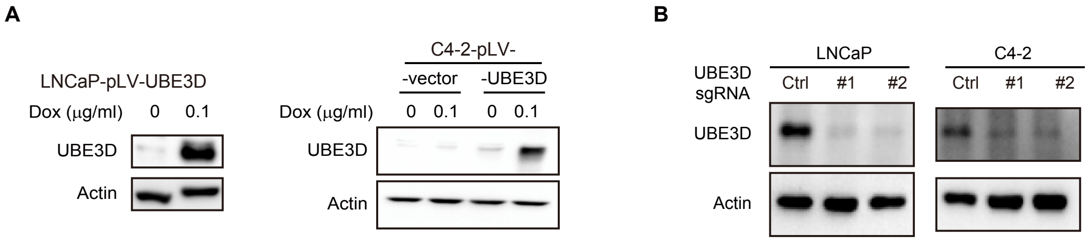
A
LNCaP-pLV-UBE3D
Dox (μg/ml)
0
0.1
UBE3D
Actin
C4-2-pLV-
-vector
-UBE3D
Dox (μg/ml)
0
0.1
0
0.1
UBE3D
Actin
B
LNCaP
C4-2
UBE3D sgRNA
Ctrl
#1
#2
Ctrl
#1
#2
UBE3D
Actin

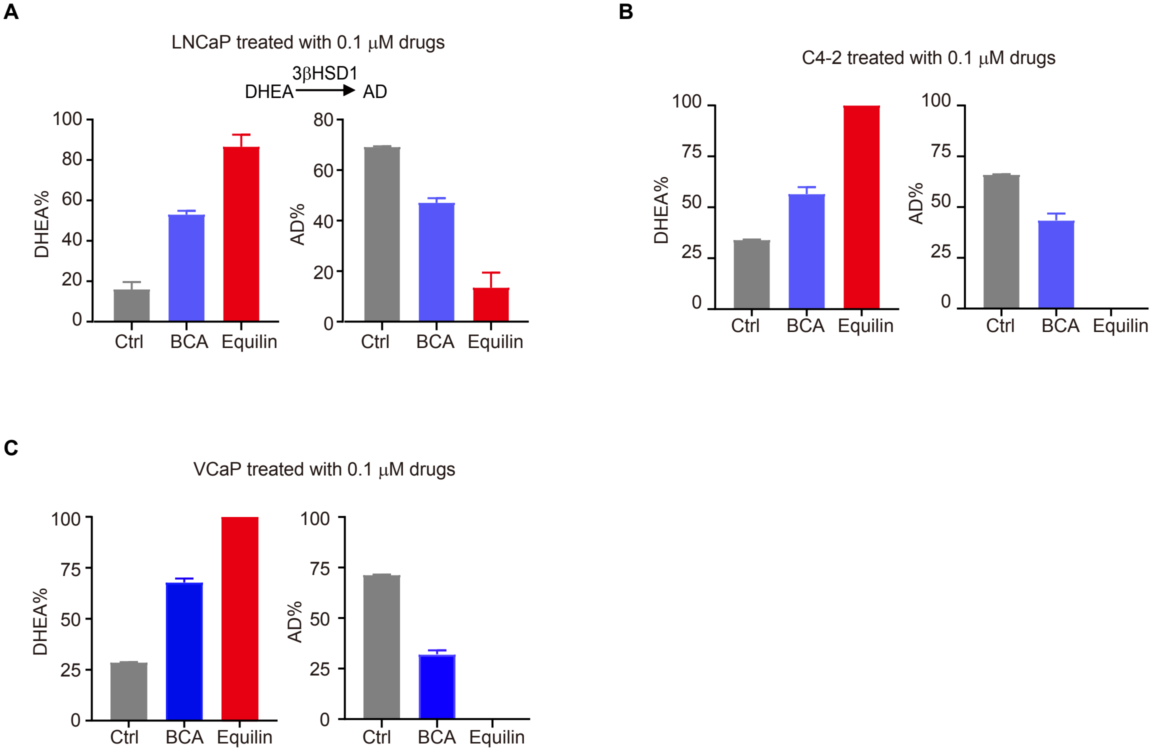
A
LNCaP treated with 0.1 μM drugs
3βHSD1
DHEA
AD
100
80
60
40
20
0
DHEA%
Ctrl
BCA
Equilin
80
60
40
20
0
AD%
Ctrl
BCA
Equilin
B
C4-2 treated with 0.1 μM drugs
100
75
50
25
0
DHEA%
Ctrl
BCA
Equilin
100
75
50
25
0
AD%
Ctrl
BCA
Equilin
C
VCaP treated with 0.1 μM drugs
100
75
50
25
0
DHEA%
Ctrl
BCA
Equilin
100
75
50
25
0
AD%
Ctrl
BCA
Equilin

BCA docking with 3βHSD1
(SP: -8.24 kcal/mol; XP: -9.07 kcal/mol)

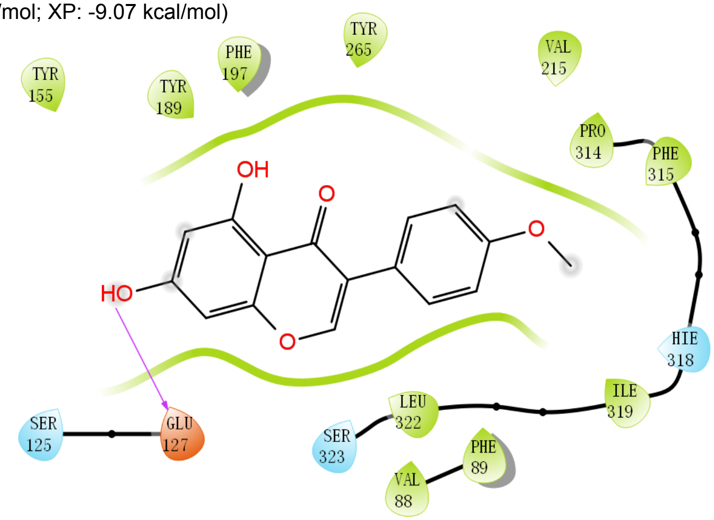

DHEA docking with 3βHSD1
(SP: -9.55 kcal/mol; XP: -10.47 kcal/mol)

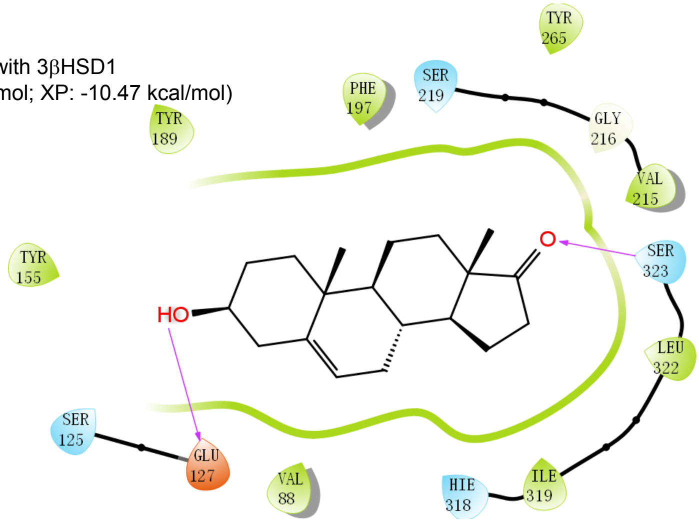

Equilin docking with 3βHSD1
(SP: -9.00 kcal/mol; XP: -10.92 kcal/mol)

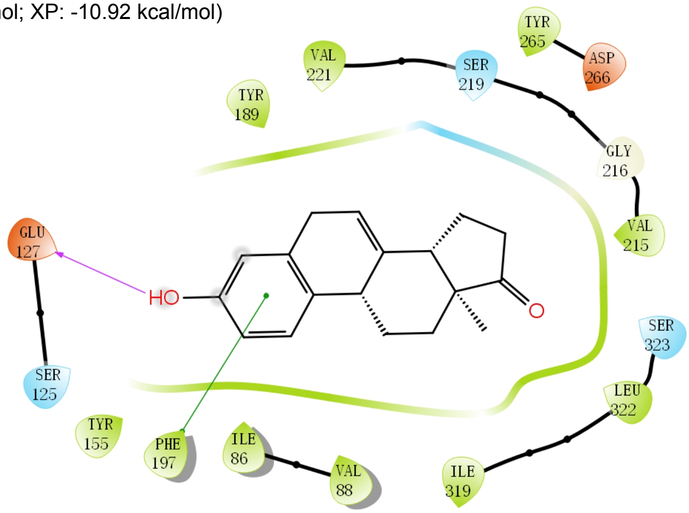

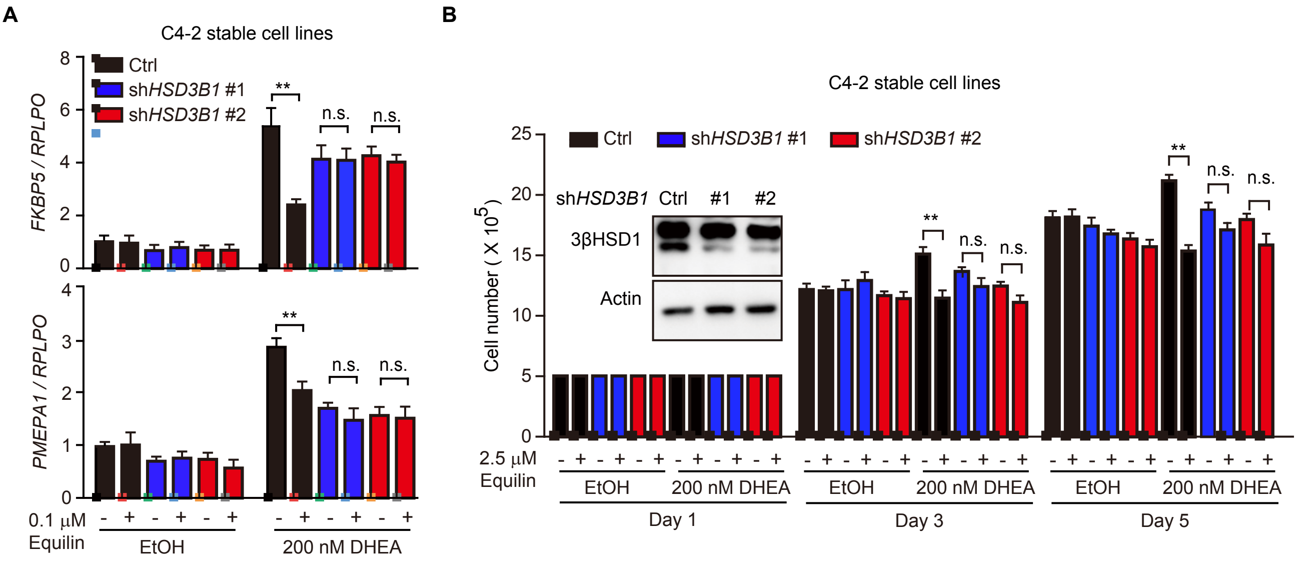
A
C4-2 stable cell lines
Ctrl
shHSD3B1 #1
shHSD3B1 #2
FKBP5 / RPLPO
PMEPA1 / RPLPO
**
n.s.
0.1 μM Equilin
EtOH
200 nM DHEA
B
C4-2 stable cell lines
Cell number (X 10^5)
shHSD3B1 Ctrl #1 #2
3βHSD1
Actin
2.5 μM Equilin
Day 1
Day 3
Day 5

Figure legends for supplemental figures

Supplemental Figure 1. Finasteride shows no direct effect on 3βHSD1 activity. (A) Percentage of androstenedione in biopsies collected from different patients. AD, androstenedione. **(B)** Effects of finasteride and dutasteride on DHEA utilization in LNCaP cells. [^{3}H]-DHEA was used to treat LNCaP cells together with or without finasteride and dutasteride. **(C)** Effect of dutasteride on DHEA utilization in biopsies. The same biopsy sample was divided into two groups with the same weight and treated with [^{3}H]-DHEA together with or without dutasteride. A total of 24 biopsies were collected. Dut, dutasteride. Lines indicate the median and interquartile range. Student's t test. **, $P < 0.01$. Results are shown as mean ± SD.

Supplemental Figure 2. Characterization of GWAS signature. (A) Correlations of germline variants and gene expression in GTEx prostate tissue. **(B)** Correlations of GWAS signature and *HSD3B1* genotype in UKBB database. 1-way ANOVA. *, $P < 0.05$; **, $P < 0.01$.

Supplemental Figure 3. Somatic mutations in our cohort. (A) Molecular landscape of somatic mutations in our cohort. Each column represents an individual biopsy. Specific molecular profiles were subsequently shown: top mutated genes, variant types, mutation signature, CNV length. (**B**) Average mutation frequency in patients at different disease stages. Alteration frequency is the average clonal abundance of a mutated gene over all biopsies within each metabolic group. (**C** and **D**) Alteration

frequency (G-score) of amplified and deleted genes in patients at different disease stages.

Supplemental Figure 4 Differentially expressed genes in biopsies with distinct prostatic DHEA utilization activities. (A) Heatmap of differentially expressed genes in biopsies with high or low metabolic activity. **(B)** Enriched pathways in biopsies with high metabolic activity.

Supplemental Figure 5. Clinical relevance of DHEA transcriptomic signature in TCGA. Correlations of the DHEA transcriptomic signature with tumor stage, Gleason score, and lymph node metastasis were analyzed in TCGA database. The DHEA transcriptomic signature scores for each patient in TCGA were calculated. 1-way ANOVA.

Supplemental Figure 6. Prediction performance of individual transcriptomic signature genes. Random grouping was performed over 200 times by selecting 14 biopsies with high metabolic activity and 10 biopsies with low metabolic activity from the 60 sequenced biopsies. The specificity and sensitivity of each gene from the DHEA transcriptomic signature were evaluated by random forest algorithm.

Supplemental Figure 7. Clinical relevance of potential 3βHSD1 ubiquitin ligases. (A) Expression of ubiquitin ligases in biopsies showing high or low metabolic activity in our cohort. **(B)** Correlation of ubiquitin ligases with treatment duration in TCGA database. Patients in TCGA database received prostatectomy and biochemical recurrence was

determined. **(C)** Effect of ubiquitin ligases on 3βHSD1 abundance. Ubiquitin ligases were individually knocked down in LNCaP cells. Student's t test. **, $P < 0.01$.

Supplemental Figure 8. UBE3D facilitates 3βHSD1 ubiquitylation. (A) Proteasome inhibitors suppress UBE3D-mediated 3βHSD1 degradation. UBE3D and 3βHSD1 were overexpressed in HEK 293T cells. MG132, proteasome inhibitor, 10 μM; PS341, proteasome inhibitor, 1 μM; Baf-1, lysosome inhibitor, 100 nM. **(B)** Half-life of endogenous 3βHSD1 in VCaP cells with or without UBE3D knockdown. CHX, cycloheximide, 100 μM. **(C)** HECT domain of UBE3D is essential for 3βHSD1 ubiquitylation. UBE3D or UBED with HECT domain deleted was overexpressed in HEK 293T cells together with 3βHSD1. MG132, 10 μM. **(D)** Protein samples for mass spectrometry obtained from an in vivo ubiquitination assay in HEK 293T cells. **(E)** K55 in 3βHSD1 is essential for UBE3D-mediated ubiquitylation. Wildtype 3βHSD1 or 3βHSD1 (K55R) mutant was transfected into HEK 293T cells together with UBE3D. (**F**) Effects of UBE3D on stabilities different 3βHSD1 mutants. N isoform, 3βHSD1 (367N) isoform; T isoform, 3βHSD1 (367T) isoform.

Supplemental Figure 9. Generations of stable cell lines. (A) Doxycycline (Dox)-induced UBE3D expressing cells in LNCaP and C4-2 cells. **(B)** Stable cells with UBE3D knocked out in LNCaP and C4-2 cells.

Supplemental Figure 10. **Equilin inhibits 3βHSD1.** Equilin inhibits 3βHSD1 more potently than biochanin-A (BCA) in LNCaP (A), C4-2 (B), and VCaP (C) cells. [^{3}H]-DHEA was used to treat VCaP cells with equilin and BCA.

Supplemental Figure 11. Docking model of equilin, BCA and DHEA with 3βHSD1. The 3βHSD1 structure was built according to AlphaFold2. SP, standard precision mode; XP, extra-precision mode.

Supplemental Figure 12. 3βHSD1 is required for equilin to inhibit prostate cancer development. (A) Equilin showed limited effects on the expression of AR target genes in 3βHSD1-depelted C4-2 stable cell lines. **(B)** Equilin showed limited effects on cell proliferation in 3βHSD1-depelted C4-2 stable cell lines. Results are shown as mean ± SD. *, $P < 0.05$; **, $P < 0.01$ by Student's t test.

Supplemental Table 1. Characteristics of patients in Figure 1D.

Supplemental Table 2. Baseline characteristics of patients receiving ADT in Figure 1 E and F.

Supplemental Table 3. Characteristics of patients in Figure 1G.

Supplemental Table 4. Uncommon variants associated with prostatic DHEA utilization.

Supplemental Table 1. Characteristics of patients in Figure 1D.

Biopsy number	3βHSD1 activity	Oxidized DHEA	DHEA	Potent_androgen	Tissue anatomy	Cancer cells identified in biopsy	Treatment	Disease stage	Age	PSA	Gleason score (biopsy)	Gleason score (patient)	TNM
P105-3-DHEA-84H	30.28	14.36	59.71	25.93	Peripheral zone	No	naive	Benign	63	8.84	/	/	/
P105-6-DHEA-84H	46.37	27.9	38.67	33.44	Transition zone	No	naive	Benign	63	8.84	/	/	/
P50-3-DHEA-84H	0.00	13.01	86.99	0	Peripheral zone	No	naive	Benign	69	41	/	/	/
P50-5-DHEA-84H	7.12	12.03	81.71	6.26	Transition zone	No	naive	Benign	69	41	/	/	/
P114-8-DHEA-84H	44.01	27.82	40.42	31.77	Peripheral zone	No	naive	Benign	67	11	/	/	/
P122-3-DHEA-84H	50.95	24.67	36.95	38.38	Peripheral zone	No	naive	Localized prostate cancer	63	20	/	3+3=6	T2N0M0
P122-6-DHEA-84H	34.02	24.76	49.64	25.6	Transition zone	Yes	naive	Localized prostate cancer	63	20	3+3=6	3+3=6	T2N0M0
P129-1-DHEA-84H	13.53	17.95	70.96	11.1	Peripheral zone	/	naive	Localized prostate cancer	63	20	/	4+3=7	T2N0M0
P129-6-DHEA-84H	31.86	17.91	55.94	26.15	Transition zone	/	naive	Localized prostate cancer	63	20	/	4+3=7	T2N0M0
P84-4-DHEA-84H	17.81	16.78	68.41	14.82	Peripheral zone	Yes	naive	Localized prostate cancer	53	30.4	4+5=9	4+5=9	T2N0M0
P84-7-DHEA-84H	0.00	8.87	91.13	0	Peripheral zone	Yes	naive	Localized prostate cancer	53	30.4	4+5=9	4+5=9	T2N0M0
P126-3-DHEA-84H	35.37	29.21	45.75	25.04	Peripheral zone	No	naive	Benign	62	5.34	/	/	/
P126-5-DHEA-84H	49.88	28.86	35.65	35.48	Transition zone	No	naive	Benign	62	5.34	/	/	/
P145-5-DHEA-84H	60.12	13.5	34.5	52	Transition zone	No	naive	Benign	58	8.77	/	/	/
P44-7-DHEA-84H	45.08	22.01	42.83	35.16	Peripheral zone	No	naive	Localized prostate cancer	68	12	/	3+3=6	T2N0M0
P262-3-DHEA-84H	46.82	34.82	34.67	30.52	Peripheral zone	No	naive	Benign	47	7.64	/	/	/
P262-5-DHEA-84H	43.10	23.69	43.42	32.89	Transition zone	No	naive	Benign	47	7.64	/	/	/
P255-5-DHEA-84H	77.87	25.67	16.45	57.88	Transition zone	Yes	naive	Metastatic prostate cancer	63	87.51	4+3=7	4+3=7	T2N0M1

P255-7-DHEA-84H	50.64	22.07	38.47	39.46	Peripheral zone	Yes	naive	Metastatic prostate cancer	63	87.51	4+3=7	4+3=7	T2N0M1
P53-3-DHEA-84H	42.72	19.58	46.05	34.35	Peripheral zone	No	naive	Benign	68	41	/	/	/
P53-9-DHEA-84H	36.97	21.65	49.38	28.96	Transition zone	No	naive	Benign	68	41	/	/	/
P207-5-DHEA-84H	13.89	21.31	67.76	10.93	Transition zone	/	naive	Localized prostate cancer	74	81.83	/	3+4=7	T3N0M0
P152-2-DHEA-84H	15.59	16.94	70.11	12.95	Peripheral zone	No	naive	Benign	59	34.63	/	/	/
P152-6-DHEA-84H	5.59	8.26	86.61	5.13	Peripheral zone	No	naive	Benign	59	34.63	/	/	/
P52-YOUWAI-DHEA-84H	45.53	14.03	46.83	39.14	/	/	naive	Localized prostate cancer	70	6.27	/	3+3=6	T1N0M0
P218-3-DHEA-84H	21.19	24.58	59.44	15.98	Peripheral zone	No	naive	Benign	87	11.6	/	/	/
P218-5-DHEA-84H	36.21	20.07	50.98	28.94	Transition zone	No	naive	Benign	87	11.6	/	/	/
P248-2-DHEA-84H	35.97	21.74	50.12	28.15	Peripheral zone	Yes	naive	Localized prostate cancer	68	15.71	4+4=8	4+4=8	T2N0M0
P248-5-DHEA-84H	32.44	12.86	58.88	28.27	Transition zone	Yes	naive	Localized prostate cancer	68	15.71	4+4=8	4+4=8	T2N0M0
P54-10-DHEA-84H	46.60	35.21	34.6	30.19	Peripheral zone	No	naive	Benign	80	14.28	/	/	/
P54-3-DHEA-84H	27.03	10.79	65.1	24.12	Peripheral zone	No	naive	Benign	80	14.28	/	/	/
P27-7-DHEA-84H	39.27	11.53	53.72	34.73	Peripheral zone	No	naive	Benign	60	21.4	/	/	/
P61-2-DHEA-84H	66.57	22.61	25.87	51.52	Peripheral zone	No	naive	Benign	77	50	/	/	/
P61-5-DHEA-84H	67.35	28.89	23.22	47.89	Transition zone	No	naive	Benign	77	50	/	/	/
P231-3-DHEA-84H	36.05	40.2	38.24	21.56	Peripheral zone	No	naive	Benign	72	11.63	/	/	/
P231-5-DHEA-84H	46.49	31.61	36.6	31.8	Transition zone	No	naive	Benign	72	11.63	/	/	/
P153-3-DHEA-84H	39.89	21.26	47.34	31.41	Peripheral zone	Yes	naive	Localized prostate cancer	71	18.9	3+3=6	3+3=6	T2N0M0
P121-3-DHEA-84H	0.00	9.33	90.67	0	Peripheral zone	No	naive	Benign	55	5.85	/	/	/

P202-10-DHEA-84H	44.75	19.38	44.54	36.08	Transition zone	No	naive	Localized prostate cancer	67	6.5	/	3+3=6	T2N0M0
P233-2-DHEA-84H	34.66	38.23	40.37	21.41	Peripheral zone	Yes	naive	Metastatic prostate cancer	44	64.74	/	/	T4N1M1
P233-4-DHEA-84H	25.58	21.26	58.59	20.14	Peripheral zone	Yes	naive	Metastatic prostate cancer	44	64.74	/	/	T4N1M1
P43-3-DHEA-84H	70.58	31.72	20.09	48.2	Peripheral zone	Yes	naive	Metastatic prostate cancer	43	74	4+5=9	4+5=9	T4N1M1
P43-7-DHEA-84H	29.39	19.03	57.17	23.8	Peripheral zone	Yes	naive	Metastatic prostate cancer	43	74	4+4=8	4+5=9	T4N1M1
P60-10-DHEA-84H	56.50	31.54	29.78	38.68	Transition zone	No	naive	Benign	52	4.9	/	/	/
P60-7-DHEA-84H	55.77	26.97	32.3	40.73	Peripheral zone	No	naive	Benign	52	4.9	/	/	/
P110-10-DHEA-84H	48.07	33.02	34.78	32.19	Transition zone	No	naive	Localized prostate cancer	57	9.1	/	3+3=6	T2N0M0
P110-3-DHEA-84H	53.03	23.45	35.95	40.59	Peripheral zone	Yes	naive	Localized prostate cancer	57	9.1	3+3=6	3+3=6	T2N0M0
P70-3-DHEA-84H	4.47	7.1	88.75	4.15	Peripheral zone	No	naive	Benign	80	13	/	/	/
P15-9-DHEA-84H	21.75	22.97	60.28	16.76	Peripheral zone	No	naive	Benign	72	8.74	/	/	/
P58-4-DHEA-84H	65.11	27.96	25.13	46.89	Transition zone	No	naive	Localized prostate cancer	72	47.5	/	4+4=8	T2N0M0
P58-6-DHEA-84H	15.43	10.46	75.72	13.82	Peripheral zone	Yes	naive	Localized prostate cancer	72	47.5	4+3=7	4+4=8	T2N0M0
P136-4-DHEA-84H	33.75	35.46	42.75	21.78	Peripheral zone	No	naive	Localized prostate cancer	65	14.76	/	3+3=6	T2N0M0
P136-5-DHEA-84H	30.87	25.76	51.32	22.92	Transition zone	No	naive	Localized prostate cancer	65	14.76	/	3+3=6	T2N0M0
P155-10-DHEA-84H	2.76	9.16	88.33	2.51	Transition zone	/	naive	Localized prostate cancer	65	4.8	/	3+4=7	T2N0M0
P155-8-DHEA-84H	11.81	18.61	71.78	9.61	Peripheral zone	/	naive	Localized prostate cancer	65	4.8	/	3+4=7	T2N0M0
P104-2-DHEA-84H	16.22	15.58	70.73	13.69	Peripheral zone	/	naive	Localized prostate cancer	71	8	/	3+3=6	T2N0M0
P81-10-DHEA-84H	10.51	7.48	82.8	9.72	Transition zone	No	naive	Localized prostate cancer	71	8	/	3+3=6	T2N0M0
P81-4-DHEA-84H	53.34	25.19	34.91	39.91	Peripheral zone	No	naive	Localized prostate cancer	71	8	/	3+3=6	T2N0M0

P48-10-DHEA-84H	30.25	31.86	47.53	20.61	Transition zone	No	naive	Benign	71	8.75	/	/	/
P48-7-DHEA-84H	88.84	32.26	7.56	60.18	Peripheral zone	No	naive	Benign	71	8.75	/	/	/
P11-5-DHEA-84H	32.53	19.98	53.99	26.03	Transition zone	No	naive	Benign	66	5.09	/	/	/
P11-7-DHEA-84H	12.06	16.16	73.72	10.11	Peripheral zone	No	naive	Benign	66	5.09	/	/	/
P18-10-DHEA-84H	27.44	13.26	62.93	23.8	Transition zone	No	naive	Localized prostate cancer	61	15	/	4+4=8	T2N0M0
P18-8-DHEA-84H	20.04	13.58	69.1	17.32	Peripheral zone	PIN	naive	Localized prostate cancer	61	15	PIN	4+4=8	T2N0M0
P253-3-DHEA-84H	80.65	31	13.35	55.64	Peripheral zone	No	naive	Benign	54	6	/	/	/
P253-5-DHEA-84H	65.98	23.7	25.96	50.34	Transition zone	No	naive	Benign	54	6	/	/	/
P234-1-DHEA-84H	5.59	14.31	80.9	4.79	Peripheral zone	No	naive	Benign	66	14.01	/	/	/
P234-10-DHEA-84H	19.31	9.88	72.72	17.4	Transition zone	No	naive	Benign	66	14.01	/	/	/
P22-10-DHEA-84H	30.20	14.16	59.92	25.92	Transition zone	No	naive	Benign	83	6.02	/	/	/
P22-3-DHEA-84H	37.53	21.11	49.28	29.61	Peripheral zone	No	naive	Benign	83	6.02	/	/	/
P101-8-DHEA-84H	54.84	23.17	34.7	42.13	Peripheral zone	No	naive	Localized prostate cancer	64	7.08	/	4+4=8	T2N0M0
P188-8-DHEA-84H	64.10	28.55	25.65	45.8	Peripheral zone	No	naive	Benign	72	2.42	/	/	/
P263-5-DHEA-84H	45.46	15.58	46.04	38.37	Transition zone	No	naive	Benign	69	14.04	/	/	/
P263-7-DHEA-84H	44.28	18.03	45.67	36.29	/	No	naive	Benign	69	14.04	/	/	/
P65-2-DHEA-84H	25.90	13.67	63.97	22.36	Peripheral zone	Yes	naive	Metastatic prostate cancer	74	700.5	4+5=9	4+5=9	T4N1M1
P65-8-DHEA-84H	45.04	22.03	42.73	35.02	Transition zone	Yes	naive	Metastatic prostate cancer	74	700.5	4+5=9	4+5=9	T4N1M1
P89-3-DHEA-84H	28.12	26.54	52.8	20.66	Peripheral zone	No	naive	Localized prostate cancer	58	9.9	/	3+3=6	T1N0M0
P89-5-DHEA-84H	46.41	15.49	45.29	39.22	Transition zone	Yes	naive	Localized prostate cancer	58	9.9	3+3=6	3+3=6	T1N0M0

P28-5-DHEA-84H	35.62	21.26	50.69	28.05	Transition zone	No	naive	Benign	76	7.48	/	/	/
P39-6-DHEA-84H	46.01	31.86	36.79	31.35	Transition zone	No	naive	Benign	72	9.8	/	/	/
P39-9-DHEA-84H	45.20	18.4	44.72	36.88	Peripheral zone	No	naive	Benign	72	9.8	/	/	/
P49-10-DHEA-84H	41.34	22.19	45.64	32.17	Transition zone	No	naive	Benign	52	2.24	/	/	/
P49-3-DHEA-84H	51.28	22.73	37.64	39.62	Peripheral zone	No	naive	Benign	52	2.24	/	/	/
P148-2-DHEA-84H	44.39	25.83	41.24	32.92	Peripheral zone	No	naive	Metastatic prostate cancer	91	940	/	4+3=7	T4N1M1b
P148-4-DHEA-84H	44.76	19.33	44.57	36.12	Peripheral zone	Yes	naive	Metastatic prostate cancer	91	940	4+3=7	4+3=7	T4N1M1b
P142-7-DHEA-84H	47.09	32.2	35.87	31.93	Peripheral zone	Yes	naive	Localized prostate cancer	69	14	3+3=6	3+3=6	T2N0M0
P159-10-DHEA-84H	43.01	23.81	43.43	32.77	Transition zone	No	naive	Benign	63	20.09	/	/	/
P159-3-DHEA-84H	17.47	11.2	73.28	15.51	Peripheral zone	No	naive	Benign	63	20.09	/	/	/
P10-WAI1-DHEA-84H	20.96	10.85	70.47	18.69	/	/	naive	Benign	75	2.03	/	/	/
P10-WAI2-DHEA-84H	9.94	11.82	79.42	8.77	/	/	naive	Benign	75	2.03	/	/	/
P10-YI1-DHEA-84H	13.91	6.6	80.42	12.99	/	/	naive	Benign	75	2.03	/	/	/
P175-5-DHEA-84H	25.55	14.94	63.33	21.73	Transition zone	No	naive	Localized prostate cancer	78	17.59	/	3+3=6	T1N0M0
P232-4-DHEA-84H	44.61	26.68	40.61	32.7	Transition zone	Yes	naive	Metastatic prostate cancer	72	302	3+4=7	3+4=7	T4N1M1
P232-6-DHEA-84H	47.63	22.66	40.5	36.84	Peripheral zone	Yes	naive	Metastatic prostate cancer	72	302	3+4=7	3+4=7	T4N1M1
P239-YOUWAI-DHEA-84H	46.74	16.06	44.7	39.23	/	/	naive	Benign	51	NA	/	/	/
P239-YOUYI-DHEA-84H	25.66	11.56	65.75	22.69	/	/	naive	Benign	51	NA	/	/	/
P13-2-DHEA-84H	60.53	15.7	33.27	51.02	Peripheral zone	Yes	naive	Metastatic prostate cancer	76	100	4+4=8	4+4=8	T2N0M1
P13-5-DHEA-84H	62.19	25.14	28.31	46.56	Transition zone	Yes	naive	Metastatic prostate cancer	76	> 100	4+4=8	4+4=8	T2N0M1

P260-5-DHEA-84H	30.60	16.94	57.64	25.41	Transition zone	No	naive	Metastatic prostate cancer	67	513	/	4+4=8	T4N1M1
P260-7-DHEA-84H	49.91	30.53	34.8	34.68	Peripheral zone	Yes	naive	Metastatic prostate cancer	67	513	4+4=8	4+4=8	T4N1M1
P26-10-DHEA-84H	43.79	20.56	44.66	34.79	Transition zone	Yes	naive	Localized prostate cancer	60	24.95	3+3=6	3+3=6	T2N0M0
P26-3-DHEA-84H	45.69	20.89	42.96	36.14	Peripheral zone	Yes	naive	Localized prostate cancer	60	24.95	3+3=6	3+3=6	T2N0M0
P130-3-DHEA-84H	46.73	41.28	31.28	27.44	Peripheral zone	Yes	naive	Benign	68	44.2	4+4=8	4+4=8	T3N1M0
P130-8-DHEA-84H	15.39	19.87	67.81	12.33	Peripheral zone	Yes	naive	Benign	68	44.2	4+4=8	4+4=8	T3N1M0
P242-3-DHEA-84H	52.79	19.06	38.22	42.73	Peripheral zone	No	naive	Benign	68	12.57	/	/	/
P242-5-DHEA-84H	43.35	18.03	46.44	35.54	Transition zone	No	naive	Benign	68	12.57	/	/	/
P92-2-DHEA-84H	36.87	31.08	43.51	25.41	Peripheral zone	No	naive	Benign	60	42.5	/	/	/
P92-5-DHEA-84H	33.53	22.36	51.61	26.03	Transition zone	No	naive	Benign	60	42.5	/	/	/
P154-1-DHEA-84H	15.45	14.85	72	13.16	Peripheral zone	Yes	naive	Metastatic prostate cancer	69	16.38	4+3=7	4+3=7	T2N0M1b
P154-5-DHEA-84H	9.98	15.01	76.51	8.48	Transition zone	No	naive	Metastatic prostate cancer	69	16.38	/	4+3=7	T2N0M1b
P246-3-DHEA-84H	0.00	4.96	95.04	0	Peripheral zone	Yes	naive	Localized prostate cancer	70	70	4+3=7	4+3=7	T2N0M0
P246-5-DHEA-84H	8.54	7.29	84.78	7.92	Transition zone	Yes	naive	Localized prostate cancer	70	70	4+3=7	4+3=7	T2N0M0
P45-10-DHEA-84H	64.28	33.93	23.6	42.47	Transition zone	No	naive	Benign	67	24	/	/	/
P45-7-DHEA-84H	44.93	33.27	36.76	29.99	Peripheral zone	No	naive	Benign	67	24	/	/	/
P20-A-DHEA-84H	35.37	11.25	57.35	31.38	/	/	naive	Benign	63	0.68	/	/	/
P63-3-DHEA-84H	38.18	21.06	48.79	30.13	Peripheral zone	Yes	naive	Metastatic prostate cancer	82	875.8	4+5=9	4+5=9	T4N1M1
P63-8-DHEA-84H	91.94	34.97	5.24	59.79	Transition zone	Yes	naive	Metastatic prostate cancer	82	875.8	4+3=7	4+5=9	T4N1M1
P102-7-DHEA-84H	46.82	35.79	34.15	30.07	Peripheral zone	No	naive	Benign	76	17.9	/	/	/

P79-10-DHEA-84H	40.19	15.07	50.8	34.13	Transition zone	No	naive	Benign	64	NA	/	/	/
P79-2-DHEA-84H	55.80	18.34	36.09	45.57	Peripheral zone	No	naive	Benign	64	NA	/	/	/
P51-5-DHEA-84H	35.03	25.83	48.19	25.98	Transition zone	No	naive	Benign	54	11.46	/	/	/
P51-9-DHEA-84H	55.51	27.29	32.35	40.37	Peripheral zone	No	naive	Benign	54	11.46	/	/	/
P116-1-DHEA-84H	63.57	30	25.5	44.5	Peripheral zone	Yes	naive	Localized prostate cancer	59	38	4+4=8	4+4=8	T2N0M0
P116-2-DHEA-84H	77.82	29.89	15.55	54.55	Peripheral zone	Yes	naive	Localized prostate cancer	59	38	4+4=8	4+4=8	T2N0M0
P264-4-DHEA-84H	52.87	15.71	39.73	44.57	Transition zone	No	naive	Localized prostate cancer	60	11.17	/	4+3=7	T2N0M0
P264-6-DHEA-84H	64.97	24.21	26.55	49.24	Peripheral zone	No	naive	Localized prostate cancer	60	11.17	/	4+3=7	T2N0M0
P66-2-DHEA-84H	55.62	35.5	28.63	35.88	Peripheral zone	Yes	naive	Localized prostate cancer	73	26.5	4+3=7	4+3=7	T2N0M0
P66-8-DHEA-84H	47.41	27.11	38.33	34.56	Transition zone	No	naive	Localized prostate cancer	73	26.5	/	4+3=7	T2N0M0
P256-5-DHEA-84H	80.34	27.21	14.31	58.48	Transition zone	No	naive	Localized prostate cancer	75	12.03	/	3+3=6	T2N0M0
P256-7-DHEA-84H	71.68	25.54	21.09	53.38	Peripheral zone	No	naive	Localized prostate cancer	75	12.03	/	3+3=6	T2N0M0
P224-1-DHEA-84H	0.00	13.67	86.33	0	Peripheral zone	Yes	naive	Localized prostate cancer	58	13.54	4+4=8	4+4=8	T2N0M0
P224-5-DHEA-84H	26.23	20.52	58.63	20.85	Transition zone	Yes	naive	Localized prostate cancer	58	13.54	4+4=8	4+4=8	T2N0M0
P62-12-DHEA-84H	37.30	14.16	53.82	32.02	Transition zone	No	naive	Benign	81	28.08	/	/	/
P62-9-DHEA-84H	29.76	16.34	58.76	24.9	Peripheral zone	No	naive	Benign	81	28.08	/	/	/
P201-4-DHEA-84H	58.77	12.91	35.91	51.18	Transition zone	Yes	naive	Metastatic prostate cancer	84	144	4+3=7	4+4=8	T4N1M1
P201-6-DHEA-84H	49.74	15.99	42.22	41.79	Peripheral zone	Yes	naive	Metastatic prostate cancer	84	144	4+4=8	4+4=8	T4N1M1
P193-10-DHEA-84H	41.69	17.35	48.19	34.46	Transition zone	Yes	naive	Localized prostate cancer	63	20.66	3+4=7	3+4=7	T1N0M0
P193-3-DHEA-84H	58.27	30.36	29.06	40.58	Peripheral zone	No	naive	Localized prostate cancer	63	20.66	/	3+4=7	T1N0M0

P112-3-DHEA-84H	23.77	21.75	59.65	18.6	Peripheral zone	No	naive	Benign	61	20.03	/	/	/
P112-7-DHEA-84H	38.39	25.87	45.68	28.46	Peripheral zone	No	naive	Benign	61	20.03	/	/	/
P87-4-DHEA-84H	40.57	15.17	50.42	34.42	Peripheral zone	Yes	naive	Localized prostate cancer	72	NA	3+3=6	3+3=6	T2N0M0
P87-7-DHEA-84H	14.99	11.86	74.93	13.21	Peripheral zone	No	naive	Localized prostate cancer	72	NA	/	3+3=6	T2N0M0
P71-5-DHEA-84H	58.01	16.47	35.07	48.45	Transition zone	Yes	naive	Localized prostate cancer	59	12.63	3+3=6	3+3=6	T1N0M0
P71-7-DHEA-84H	76.23	30.41	16.54	53.04	Peripheral zone	Yes	naive	Localized prostate cancer	59	12.63	3+3=6	3+3=6	T1N0M0
P88-5-DHEA-84H	0.00	6.75	93.25	0	Transition zone	/	naive	Localized prostate cancer	59	12.63	/	3+4=7	T1N0M0
P25-10-DHEA-84H	23.24	28.4	54.95	16.64	Transition zone	No	naive	Localized prostate cancer	64	19.4	/	4+4=8	T2N0M0
P25-7-DHEA-84H	49.87	15.06	42.58	42.36	Peripheral zone	No	naive	Localized prostate cancer	64	19.4	/	4+4=8	T2N0M0
P221-YOUWAI-DHEA-84H	46.20	15.59	45.41	39	/	/	naive	Benign	77	2.83	/	3+3=6	/
P221-YOUYI-DHEA-84H	18.94	13.38	70.22	16.41	/	/	naive	Benign	77	2.83	/	3+3=6	/
P90-2-DHEA-84H	9.67	14.82	76.94	8.24	Peripheral zone	No	naive	Benign	59	9.12	/	/	/
P149-5-DHEA-84H	53.40	19.93	37.31	42.76	Transition zone	No	naive	Benign	60	13.17	/	/	/
P33-4-DHEA-84H	46.64	26.56	39.19	34.25	Transition zone	Yes	naive	Metastatic prostate cancer	77	368	4+4=8	4+4=8	T4N1M1
P33-5-DHEA-84H	69.29	22.74	23.73	53.53	Peripheral zone	Yes	naive	Metastatic prostate cancer	77	368	4+4=8	4+4=8	T4N1M1
P252-3-DHEA-84H	51.75	27.13	35.16	37.71	Peripheral zone	No	naive	Benign	62	5.33	/	/	/
P252-5-DHEA-84H	18.17	8.81	74.62	16.57	Transition zone	No	naive	Benign	62	5.33	/	/	/
P261-3-DHEA-84H	34.74	21.3	51.37	27.35	Peripheral zone	No	naive	Metastatic prostate cancer	71	161.5	/	4+4=8	T4N1M1
P261-5-DHEA-84H	78.74	25.28	15.88	58.83	Peripheral zone	No	naive	Metastatic prostate cancer	71	161.5	/	4+4=8	T4N1M1
P73-4-DHEA-84H	73.52	29.37	18.7	51.93	Transition zone	No	naive	Benign	68	12	/	/	/

P73-6-DHEA-84H	52.29	24.92	35.82	39.26	Peripheral zone	No	naive	Benign	68	12	/	/	/

Supplemental Table 2. Baseline characteristics of patients receiving ADT in Figure 1 E and F.

Patient number	Age	3βHSD1 activity	Baseline PSA	ISUP	GS score	Baseline TNM	Nadiar PSA	Treatment duration	Resistence
1	72	71.8596411	61.08	2	3+4	T3N1M1c	0.043	325	1
2	77	58.24817518	6.82	4	4+4	T4N1M1	1.802	203	1
3	44	48.23797401	12.69	5	4+5	T4N1M1	3.17	132	1
4	85	42.51598857	41.33		NA	T2N0M1b	0.003	852	1
5	78	10.87570621	252.9	2	3+4	T1N0M0	5.46	336	1
6	95	42.10526316	132		NA	T3NxM1c	0.197	896	0
7	63	24.54212454	20.1	1	3+3	T2N0M0	0.003	1253	0
8	74	46.35812283	320	3	4+3	T4N1M1	0.048	731	0
9	82	42.92127727	13.13	3	4+3	T2N0M0	0.013	640	0
10	82	18.25983178	1093	3	4+3	T4N1M1	0.006	606	0
11	81	40.5044977	9.48	1	3+3	T2N0M0	0.003	901	0
12	87	58.96041482	80.47	2	3+4	T2N0M0	0.066	538	0
13	72	54.67671458	2.82	5	4+5	T3N0M0	0.003	490	0
14	65	29.4626746	0.47	1	3+3	T2N0M0	0.003	645	0
15	70	49.70308789	2.99	2	3+4	T2N0M0	0.003	595	0
16	58	37.90593151	10.13	1	3+3	T1N0M0	0.003	818	0
17	76	47.33590734	121.6	4	4+4	T3N0M1b	0.391	267	1
18	74	56.66458723	24.63	3	4+3	T2N0Mx	0.003	672	1
19	75	37.84581672	0.52	4	4+4	T2N0M0	0.006	810	0
20	63	63.93011953	87.51	3	4+3	T2N0M1	0.015	701	0
21	75	53.25732899	39.52		NA	T2N0M0	0.006	311	0
22	75	43.00293405	32.41	3	4+3	T2N0M1	0.006	280	0
23	67	32.92194797	27.07	2	3+4	T2N0M0	0.003	738	0

Supplemental Table 3. Characteristics of patients in Figure 1G.

Prior treatment	Disease stage	Biopsy number	3βHSD1 activity	oxidized DHEA	DHEA	potent_androgen	Tissue anatomy	Cancer cells identified in biopsy	Age
finasteride	Benign	P151-4-DHEA-84H	42.12	31.81	39.47	28.72	Peripheral zone	No	82.00
finasteride	Benign	P151-7-DHEA-84H	28.34	19.62	57.60	22.78	Peripheral zone	No	82.00
finasteride	Benign	P162-2-DHEA-84H	53.84	24.40	34.90	40.70	Peripheral zone	No	80.00
finasteride	Benign	P162-7-DHEA-84H	49.60	20.49	40.07	39.44	Peripheral zone	No	80.00
finasteride	Benign	P95-1-DHEA-84H	14.24	31.30	58.91	9.78	Peripheral zone	No	72.00
finasteride	Benign	P95-4-DHEA-84H	13.73	33.28	57.56	9.16	Peripheral zone	No	72.00
finasteride	Benign	P12-4-DHEA-84H	18.96	18.29	66.22	15.49	Peripheral zone	No	63.00
finasteride	Benign	P12-5-DHEA-84H	22.17	13.97	66.95	19.07	Transition zone	No	63.00
finasteride	Benign	P329-3-DHEA-84H	10.59	17.49	73.76	8.74	Peripheral zone	No	70.00
finasteride	Benign	P329-6-DHEA-84H	19.26	13.92	69.50	16.58	Transition zone	No	70.00
finasteride	Benign	P247-6-DHEA-84H	46.61	19.37	43.05	37.58	Transition zone	No	66.00
finasteride	Benign	P247-8-DHEA-84H	61.27	20.83	30.66	48.50	Peripheral zone	No	66.00
finasteride	Benign	P174-10-DHEA-84H	29.01	17.49	58.57	23.93	Peripheral zone	No	63.00
finasteride	Benign	P174-3-DHEA-84H	30.81	43.30	39.23	17.47	Peripheral zone	No	63.00
finasteride	Benign	P217-3-DHEA-84H	7.47	7.89	85.24	6.88	Peripheral zone	No	66.00
finasteride	Benign	P217-5-DHEA-84H	35.24	28.85	46.08	25.07	Transition zone	No	66.00
finasteride	Benign	P131-5-DHEA-84H	5.15	10.05	85.32	4.63	Transition zone	No	80.00
finasteride	Benign	P131-6-DHEA-84H	24.70	19.65	60.50	19.85	Peripheral zone	No	80.00
finasteride	Localized prostate cancer	P331-WAIZHOU-DHEA-84H	31.18	18.06	56.39	25.55	/	/	69.00
finasteride	Localized prostate cancer	P331-YIXING-DHEA-84H	23.21	19.83	61.56	18.61	/	/	69.00
finasteride	Localized prostate cancer	P76-YOUWAI-DHEA-84H	11.36	13.36	76.81	9.84	/	/	70.00
finasteride	Localized prostate cancer	P76-ZUOWAI-DHEA-84H	9.01	10.42	81.51	8.07	/	/	70.00
finasteride	Localized prostate cancer	P143-5-DHEA-84H	36.91	21.91	49.27	28.83	Transition zone	Yes	61.00
finasteride	Localized prostate cancer	P143-7-DHEA-84H	25.58	21.20	58.64	20.16	Peripheral zone	No	61.00
finasteride	Localized prostate cancer	P216-5-DHEA-84H	70.84	21.46	22.90	55.64	Transition zone	No	73.00
finasteride	Localized prostate cancer	P216-8-DHEA-84H	48.69	18.15	42.00	39.85	Peripheral zone	No	73.00
finasteride	Localized prostate cancer	P134-3-DHEA-84H	59.82	26.83	29.39	43.76	Peripheral zone	Yes	87.00
finasteride	Localized prostate cancer	P134-6-DHEA-84H	23.43	15.40	64.79	19.82	Peripheral zone	Yes	87.00
tamsulosin	Benign	P34-5-DHEA-84H	55.16	34.37	29.43	36.21	Transition zone	No	85.00
tamsulosin	Benign	P34-7-DHEA-84H	42.98	47.19	30.11	22.70	Peripheral zone	No	85.00
tamsulosin	Benign	P197-5-DHEA-84H	51.14	25.48	36.41	38.11	Transition zone	No	66.00
tamsulosin	Benign	P197-8-DHEA-84H	54.64	17.10	37.61	45.30	Peripheral zone	No	66.00
tamsulosin	Benign	P225-3-DHEA-84H	37.26	21.50	49.26	29.25	Peripheral zone	No	/
tamsulosin	Benign	P225-5-DHEA-84H	44.10	22.67	43.22	34.10	Transition zone	No	/
tamsulosin	Benign	P111-10-DHEA-84H	27.23	24.97	54.60	20.43	Transition zone	No	68.00
tamsulosin	Benign	P111-6-DHEA-84H	33.80	27.28	48.14	24.58	Peripheral zone	No	68.00
tamsulosin	Benign	P128-3-DHEA-84H	33.28	22.11	51.97	25.92	Peripheral zone	No	78.00
tamsulosin	Benign	P128-8-DHEA-84H	7.63	17.08	76.58	6.33	Peripheral zone	No	78.00

tamsulosin	Benign	P23-6-DHEA-84H	72.31	29.18	19.61	51.20	Peripheral zone	No	80.00
tamsulosin	Benign	P23-9-DHEA-84H	35.10	20.66	51.49	27.85	Peripheral zone	No	80.00
tamsulosin	Benign	P265-3-DHEA-84H	55.97	23.21	33.81	42.98	Peripheral zone	No	67.00
tamsulosin	Benign	P265-5-DHEA-84H	27.98	15.48	60.86	23.65	Transition zone	No	67.00
tamsulosin	Benign	P82-10-DHEA-84H	57.61	25.62	31.53	42.85	Transition zone	No	71.00
tamsulosin	Benign	P82-3-DHEA-84H	31.53	15.53	57.83	26.63	Peripheral zone	No	71.00
tamsulosin	Benign	P222-3-DHEA-84H	42.50	26.28	42.39	31.33	Peripheral zone	No	68.00
tamsulosin	Benign	P222-5-DHEA-84H	37.82	21.47	48.83	29.70	Transition zone	No	68.00
tamsulosin	Benign	P118-4-DHEA-84H	16.79	11.14	73.94	14.92	Transition zone	No	84.00
tamsulosin	Benign	P118-5-DHEA-84H	46.45	18.46	43.67	37.88	Peripheral zone	No	84.00
tamsulosin	Benign	P165-3-DHEA-84H	28.01	66.16	24.36	9.48	Peripheral zone	No	66.00
tamsulosin	Benign	P165-5-DHEA-84H	46.23	38.80	32.91	28.29	Transition zone	No	66.00
tamsulosin	Benign	P223-5-DHEA-84H	38.49	36.94	38.79	24.27	Transition zone	No	87.00
tamsulosin	Benign	P223-7-DHEA-84H	24.39	14.89	64.35	20.76	Peripheral zone	No	87.00
tamsulosin	Benign	P17-10-DHEA-84H	51.27	26.59	35.77	37.63	Transition zone	No	68.00
tamsulosin	Benign	P17-7-DHEA-84H	21.56	11.88	69.12	19.00	Peripheral zone	No	68.00
tamsulosin	Benign	P198-3-DHEA-84H	33.35	14.89	56.73	28.39	Peripheral zone	No	67.00
tamsulosin	Benign	P198-5-DHEA-84H	45.07	21.25	43.26	35.49	Transition zone	No	67.00
tamsulosin	Benign	P141-12-DHEA-84H	48.90	23.86	38.92	37.24	Transition zone	No	77.00
tamsulosin	Benign	P141-4-DHEA-84H	4.38	10.37	85.70	3.93	Peripheral zone	No	77.00
tamsulosin	Benign	P99-3-DHEA-84H	12.76	14.94	74.20	10.85	Peripheral zone	No	81.00
tamsulosin	Localized prostate cancer	P168-1-DHEA-84H	19.59	27.48	58.31	14.21	Peripheral zone	No	82.00
tamsulosin	Localized prostate cancer	P168-5-DHEA-84H	41.91	29.43	41.00	29.58	Transition zone	No	82.00
tamsulosin	Localized prostate cancer	P161S-3-DHEA-84H	56.06	24.47	33.19	42.34	Peripheral zone	No	75.00
tamsulosin	Localized prostate cancer	P161S-8-DHEA-84H	46.39	26.77	39.26	33.97	Peripheral zone	Yes	75.00
tamsulosin	Localized prostate cancer	P183-2-DHEA-84H	28.84	20.58	56.51	22.90	Peripheral zone	Yes	53.00
tamsulosin	Localized prostate cancer	P183-7-DHEA-84H	28.60	25.54	53.17	21.30	Peripheral zone	Yes	53.00
tamsulosin	Localized prostate cancer	P36-10-DHEA-84H	61.37	15.67	32.57	51.75	Transition zone	Yes	87.00
tamsulosin	Localized prostate cancer	P36-6-DHEA-84H	56.20	26.18	32.33	41.49	Peripheral zone	No	87.00
tamsulosin	Localized prostate cancer	P56-2-DHEA-84H	45.77	22.14	42.22	35.64	Peripheral zone	Yes	81.00
tamsulosin	Localized prostate cancer	P56-8-DHEA-84H	35.45	18.88	52.36	28.75	Peripheral zone	/	81.00
tamsulosin	Localized prostate cancer	P135-2-DHEA-84H	33.55	22.24	51.68	26.09	Peripheral zone	No	62.00
tamsulosin	Localized prostate cancer	P205-3-DHEA-84H	76.82	28.13	16.66	55.21	Peripheral zone	Yes	79.00
tamsulosin	Localized prostate cancer	P205-5-DHEA-84H	59.08	26.39	30.12	43.49	Transition zone	No	79.00
tamsulosin	Metastatic prostate cancer	P97-3-DHEA-84H	61.45	24.44	29.13	46.43	Peripheral zone	Yes	85.00
tamsulosin	Metastatic prostate cancer	P97-4-DHEA-84H	74.87	31.33	17.26	51.42	Transition zone	Yes	85.00
tamsulosin	Metastatic prostate cancer	P100-3-DHEA-84H	43.91	21.06	44.28	34.67	Peripheral zone	Yes	76.00
tamsulosin	Metastatic prostate cancer	P100-6-DHEA-84H	50.87	23.56	37.56	38.89	Peripheral zone	Yes	76.00
tamsulosin	Metastatic prostate cancer	P85-4-DHEA-84H	73.71	26.02	19.45	54.52	Transition zone	Yes	68.00
tamsulosin	Metastatic prostate cancer	P85-7-DHEA-84H	75.81	15.22	20.51	64.27	Peripheral zone	Yes	68.00
terazosin	Benign	P113-3-DHEA-84H	58.47	27.02	30.31	42.67	Peripheral zone	No	75.00

terazosin	Benign	P113-5-DHEA-84H	32.33	27.64	48.96	23.39	Transition zone	No	75.00

Supplemental Table 4. Uncommon variants associated with prostatic DHEA consumption.

Gene	Variation	Chromosome	Position	Frequency in high activity biopsies (n=25)	Frequency in low activity biopsies (n=22)
ANKRD36	rs531465456	chr2	97113814	1	0
ANKRD36	rs542428195	chr2	97113818	1	0
ANKRD36	rs374398264	chr2	97113819	1	0
ANKRD36	rs768681489	chr2	97151897	1	0
ANKRD36	rs200297948	chr2	97162115	1	2
ANKRD36	rs771454086	chr2	97163286	0	1
ANKRD36	rs143614175	chr2	97163323	7	0
ANKRD36	rs200806944	chr2	97164440	1	0
ANKRD36	rs78284834	chr2	97164442	16	10
ANKRD36	rs199506401	chr2	97164455	13	6
ANKRD36	rs71209863	chr2	97167731	2	0
ANKRD36	rs201600563	chr2	97192885	3	1
ANKRD36	rs10203570	chr2	97202210	3	0
ANKRD36	rs751998840	chr2	97202219	3	0
ANKRD36	rs745923584	chr2	97202223	3	0
ANKRD36	rs76295552	chr2	97207944	0	1
PCDHB4	rs147934595	chr5	141123572	15	18
PCDHB4	rs568495228	chr5	141123595	11	18
TRBV5-5	rs530641209	chr7	142482736	10	3
TRBV5-5	rs372143164	chr7	142482789	2	0
TRBV5-5	rs541363853	chr7	142482822	4	1
TRBV5-5	rs573114484	chr7	142482823	4	1
TRBV5-5	rs776885061	chr7	142482977	11	7
OR4A8	rs539871	chr11	54683428	0	0
OR4A8	rs485548	chr11	54683770	6	22
AHNAK	rs115526077	chr11	62517373	1	0
AHNAK	rs1324453927	chr11	62520261	0	1
AHNAK	rs202195084	chr11	62520796	1	0
AHNAK	rs202102732	chr11	62521818	1	0
AHNAK	rs200444226	chr11	62522922	1	0
AHNAK	rs566144	chr11	62525410	0	0
AHNAK	rs1211282594	chr11	62526673	1	0
AHNAK	rs749619570	chr11	62526766	1	0
AHNAK	rs1478357138	chr11	62527245	6	0
AHNAK	rs1417666530	chr11	62527257	5	0

AHNAK	rs1299976938	chr11	62527269	5	0
AHNAK	rs75066541	chr11	62529990	0	1
AHNAK	rs114201858	chr11	62530287	0	1
AHNAK	rs1481393218	chr11	62533567	0	1
AHNAK	rs76611638	chr11	62535071	2	0
NPIPA5	rs527823489	chr16	15363700	0	2
NPIPA5	rs201487986	chr16	15363715	1	1
NPIPA5	rs531935972	chr16	15363718	0	2
NPIPA5	rs886037884	chr16	15363750	0	2
NPIPA5	rs148628178	chr16	15363897	0	1
NPIPA5	rs748959422	chr16	15363898	0	1
NPIPA5	rs200921708	chr16	15363907	9	17
NPIPA5	rs144896967	chr16	15363910	8	16
RBFOX3	rs112978510	chr17	79095541	1	0
RBFOX3	rs11653046	chr17	79183307	15	0
OR11H1	rs71235604	chr22	15528346	4	11
OR11H1	rs202150076	chr22	15528427	3	9
OR11H1	rs200330272	chr22	15528960	1	0
SPANXD	rs149669480	chrX	141697635	1	4
SPANXD	rs782213281	chrX	141698335	16	9
SPANXD	rs1472748823	chrX	141698344	16	9
SPANXD	rs148275129	chrX	141698355	16	8
